# An African tick flavivirus forming an independent clade exhibits unique exoribonuclease-resistant RNA structures in the genomic 3′-untranslated region

**DOI:** 10.1038/s41598-021-84365-9

**Published:** 2021-03-01

**Authors:** Hayato Harima, Yasuko Orba, Shiho Torii, Yongjin Qiu, Masahiro Kajihara, Yoshiki Eto, Naoya Matsuta, Bernard M. Hang’ombe, Yuki Eshita, Kentaro Uemura, Keita Matsuno, Michihito Sasaki, Kentaro Yoshii, Ryo Nakao, William W. Hall, Ayato Takada, Takashi Abe, Michael T. Wolfinger, Martin Simuunza, Hirofumi Sawa

**Affiliations:** 1 Hokudai Center for Zoonosis Control in Zambia, Research Center for Zoonosis Control, Hokkaido University, Sapporo, Japan; 2 Division of Molecular Pathobiology, Research Center for Zoonosis Control, Hokkaido University, Sapporo, Japan; 3 International Collaboration Unit, Research Center for Zoonosis Control, Hokkaido University, Sapporo, Japan; 4 Division of Global Epidemiology, Research Center for Zoonosis Control, Hokkaido University, Sapporo, Japan; 5 Department of Electrical and Information Engineering, Graduate School of Science and Technology, Niigata University, Niigata, Japan; 6 Department of Para-Clinical Studies, School of Veterinary Medicine, The University of Zambia, Lusaka, Zambia; 7 Africa Center of Excellence for Infectious Diseases of Humans and Animals, The University of Zambia, Lusaka, Zambia; 8 Drug Discovery and Disease Research Laboratory, Shionogi & Co., Ltd., Osaka, Japan; 9 Unit of Risk Analysis and Management, Research Center for Zoonosis Control, Hokkaido University, Sapporo, Japan; 10 Laboratory of Public Health, Faculty of Veterinary Medicine, Hokkaido University, Sapporo, Japan; 11 National Research Center for the Control and Prevention of Infectious Diseases (CCPID), Nagasaki University, Nagasaki, Japan; 12 Laboratory of Parasitology, Faculty of Veterinary Medicine, Hokkaido University, Sapporo, Japan; 13 National Virus Reference Laboratory, School of Medicine, University College Dublin, Dublin, Ireland; 14 Centre for Research in Infectious Diseases, School of Medicine, University College Dublin, Dublin, Ireland; 15 Global Virus Network, Baltimore, MD USA; 16 Department of Disease Control, School of Veterinary Medicine, The University of Zambia, Lusaka, Zambia; 17 Department of Theoretical Chemistry, University of Vienna, Vienna, Austria; 18 Research Group Bioinformatics and Computational Biology, Faculty of Computer Science, University of Vienna, Vienna, Austria

**Keywords:** Bioinformatics, Viral infection, Viral epidemiology, Viral vectors

## Abstract

Tick-borne flaviviruses (TBFVs) infect mammalian hosts through tick bites and can cause various serious illnesses, such as encephalitis and hemorrhagic fevers, both in humans and animals. Despite their importance to public health, there is limited epidemiological information on TBFV infection in Africa. Herein, we report that a novel flavivirus, Mpulungu flavivirus (MPFV), was discovered in a *Rhipicephalus muhsamae* tick in Zambia. MPFV was found to be genetically related to Ngoye virus detected in ticks in Senegal, and these viruses formed a unique lineage in the genus *Flavivirus*. Analyses of dinucleotide contents of flaviviruses indicated that MPFV was similar to those of other TBFVs with a typical vertebrate genome signature, suggesting that MPFV may infect vertebrate hosts. Bioinformatic analyses of the secondary structures in the 3′-untranslated regions (UTRs) revealed that MPFV exhibited unique exoribonuclease-resistant RNA (xrRNA) structures. Utilizing biochemical approaches, we clarified that two xrRNA structures of MPFV in the 3′-UTR could prevent exoribonuclease activity. In summary, our findings provide new information regarding the geographical distribution of TBFV and xrRNA structures in the 3′-UTR of flaviviruses.

## Introduction

The genus *Flavivirus* in the family *Flaviviridae* is comprised of more than 50 species, which can be divided into vector-borne flaviviruses, insect-specific flaviviruses (ISFVs), and no known arthropod vector flaviviruses (NKVs)^1^. Vector-borne flaviviruses are transmitted to vertebrate hosts by arthropod vectors, including ticks and mosquitos; a number of tick-borne flaviviruses (TBFVs) and mosquito-borne flaviviruses (MBFVs) cause serious illnesses, including encephalitis and hemorrhagic fevers, in humans and animals^1,2^. For instance, Yellow fever, Dengue hemorrhagic fever, West Nile encephalitis, Japanese encephalitis, Zika virus disease and tick-borne encephalitis are thought to be emerging and re-emerging vector-borne infectious diseases, and these are caused by flaviviruses which are distributed globally^2^.

To date, 12 TBFV species have been classified by the International Committee on Taxonomy of Viruses, and are classified into three groups: mammalian-TBFV group (*Tick-borne encephalitis virus*, *Louping ill virus*, *Powassan virus*, *Kyasanur Forest disease virus*, *Langat virus*, *Omsk hemorrhagic fever virus*, *Gadgets Gully virus,* and *Royal Farm virus*), seabird-TBFV group (*Meaban virus*, *Saumarez Reef virus,* and *Tyuleniy virus*), and probably-TBFV group (*Kadam virus*)^3^. Additionally, two unclassified TBFVs—Karshi virus and Kama virus—have been reported^4,5^. Alkhurma hemorrhagic fever viruses, a subtype of Kyasanur Forest disease virus, were identified in humans in Saudi Arabia in 1995^6^, and have been subsequently detected in humans and ticks in Egypt and Djibouti in 2010, respectively^7–9^. Kadam viruses classified as a probable member of the TBFV have been isolated from ticks, including *Rhipicephalus* spp. and *Amblyomma* sp., in Uganda and Kenya^10,11^; however, it remains unknown whether these viruses are related to human and/or animal diseases. In recent years, genome fragments of a highly divergent flavivirus, Ngoye virus, were detected in *Rhipicephalus evertsi evertsi* and *Rhipicephalus guilhoni* in Senegal, indicating that tick-related flaviviruses exhibit large heterogeneity^12^. However, there is no information on the pathogenicity and prevalence of Ngoye virus in Africa. Therefore, we set out to screen flaviviruses in ticks, including *Rhipicephalus* spp., in Zambia.

Flaviviruses are enveloped, positive-sense single-stranded RNA viruses with a genome size of approximately 9–11 kb, consisting of a 5′-untranslated region (UTR), a single open reading frame (ORF), and a 3′-UTR^1^. The ORF encodes a single polyprotein composed of three structural proteins, namely the capsid (C), pre-membrane/membrane (prM) and envelop (E) proteins, and seven non-structural (NS) proteins: NS1, NS2A, NS2B, NS3, NS4A, NS4B, and NS5^1^. These viral proteins are cleaved and matured from polyproteins by viral and host proteases^13,14^. The 5′-UTR of flaviviruses is ~ 100 nucleotides in length, and the 3′-UTR is longer than 5′-UTR (400–700 nucleotides)^15^. Characteristic secondary structures of both the 5′-UTR and the 3′-UTR are required for genome cyclization, and interaction of the UTRs is essential for viral replication and translation^16–22^. During flavivirus infection, shorter subgenomic flavivirus RNAs (sfRNAs) are produced in addition to viral genomic RNAs^23–28^. The production of sfRNA is common to all flaviviruses, and is associated with viral pathogenicity, host adaptation, and immune evasion^26–29^. These sfRNAs originate from the 3′-UTR of the flavivirus genome and result from partial degradation of viral genomic RNA by the cellular 5′-3′ exoribonuclease Xrn1, which degrades 5′-monophosphorylated RNAs in the cytoplasm^23^. Mechanistically, sfRNAs are produced by blocking the progression of Xrn1 from the 5′ end at specifically structured RNA elements in the 3′-UTR, which are termed exoribonuclease-resistant RNAs (xrRNAs). Interestingly, the secondary structures of TBFV xrRNA differ from those of MBFV xrRNA^24^. ISFV xrRNA structures are conserved among the ISFV group and diverge from MBFV xrRNA structures^30^, suggesting that classification of the flavivirus group according to these structural features may be of value. Although MBFV xrRNA structures are relatively well-characterized, limited experimental data is available on the xrRNA structures of other flaviviruses, including TBFV, ISFV, and NKV^24^. Therefore, functional characterization of secondary structures in 3′-UTRs is necessary to understand both the evolutionary history of flavivirus groups and viral adaptation to both arthropod vectors and vertebrate hosts.

Continued surveillance of TBFVs will provide key insights into the evolution of flaviviruses as well as vector/host relationship and adaptation. In this study, we attempted to detect TBFVs in field-collected ticks in Zambia. Herein, we describe the characterization of a novel flavivirus called Mpulungu flavivirus (MPFV), which was discovered in a *Rhipicephalus muhsamae* tick in Zambia and appears to be closely related to Ngoye virus. Complete genome sequencing, including the 5′- and 3′-UTRs, was conducted and analyzed for predicting host range. We have predicted and characterized the secondary structures of MPFV in the UTRs by bioinformatic analyses. Thereafter, we also clarified the xrRNA structures, which prevent exoribonuclease digestion, in the 3′-UTR by biochemical approaches.

## Results

### Discovery of a novel flavivirus in a *Rhipicephalus muhsamae* in Zambia

To identify potential tick-borne pathogens in Zambia, ticks were collected from different areas in Zambia (i.e., Isoka, Mpulungu, and Samfya). To specifically examine flaviviruses in ticks, we screened a total of 573 individual ticks for detection of flaviviruses by reverse transcription-PCR (RT-PCR) using pan-flavivirus primers (see Supplementary Tables S1, S2 online). Of these, a single flavivirus RNA genome was detected from *Rhipicephalus muhsamae* collected in a pasture in Mpulungu; the resultant RT-PCR product (~ 270 bp) was sequenced and analyzed using BLAST query (https://blast.ncbi.nlm.nih.gov/Blast.cgi). Sequencing demonstrated an 83.2% nucleotide identity with the Ngoye virus detected from *Rhipicephalus evertsi evertsi* and *Rhipicephalus guilhoni* in Senegal^12^. We attempted to isolate detected flavivirus from tick homogenates using intracerebral injections into neonatal mice and various cell lines, including African green monkey kidney (Vero E6), baby hamster kidney (BHK-21), *Ixodes scapularis* (ISE6), and *Aedes albopictus* (C6/36) cells. Several blind passages in neonatal murine brain and the aforementioned cell lines were performed; however, viral replication was not detected in either the brains of inoculated mice or passaged cell lines.

Viral genomic sequences contained in flavivirus-positive tick lysates were examined using next generation sequencing. A consensus flavivirus genome (10,596 nucleotides) was determined via bioinformatic analyses of the obtained nucleotide sequences. Based on the consensus sequence, rapid amplification of cDNA ends (RACE) was performed to determine the 5′- and the 3′-terminal sequences. The viral genome derived from *Rhipicephalus muhsamae* tick lysate was 10,868 nucleotides in length with a GC content of 56.5%. BLAST analysis indicated that this detected flavivirus appeared to be most closely related to Ngoye virus with 83.7% nucleotide identity, even though the genomic sequence of Ngoye virus was only partially available in National Center for Biotechnology Information (NCBI) databank (4176 nucleotides). This newly detected flavivirus was tentatively designated as Mpulungu flavivirus (MPFV). The determined genomic sequence of MPFV was deposited in the DNA Data Bank of Japan (DDBJ) under the accession number LC582740.

### Prediction of the ORF sequence of MPFV and phylogenetic analysis

The predicted complete ORF sequence of MPFV was 10,206 nucleotides encoding a 3401 amino acid polyprotein, and the 5′- and 3′-UTRs were 136 and 526 nucleotides in length, respectively (Table 1). The cleavage sites of both MPFV and representative flaviviruses were deduced (see Supplementary Table S3 online), and an identity comparison analysis based on amino acid sequence of each viral protein was conducted (Table 2). Identities based on the complete polyprotein sequences between MPFV and other representative flaviviruses—including TBFVs, MBFVs, ISFVs, and NKVs—were less than 45.0%. Similar results were obtained by comparative analyses based on NS3, NS4A, NS4B, and NS5 proteins. Other MPFV non-structural proteins (NS1, NS2A, NS2B, and NS3) and the structural proteins (C, PrM, and E) were also highly divergent from known flaviviruses. However, the partial polyprotein of MPFV shared a high sequence identity (95.0%) with Ngoye virus (Table 2).

**Table 1:** Genome organization of Mpulungu flavivirus.

Gene	Protein	Genome position	Length (nucleotide)	Length (amino acid)
5′-UTR	–	1–136	136	–
ORF	Polyprotein	137–10,342	10,206	3401
	C	137–385	249	83
	CTHD	386–439	54	18
	PrM	440–931	492	164
	M	707–931	225	75
	E	932–2419	1488	496
	NS1	2420–3463	1044	348
	NS2A	3464–4162	699	233
	NS2B	4163–4567	405	135
	NS3	4568–6421	1854	618
	NS4A	6422–6799	378	126
	2K	6800–6868	69	23
	NS4B	6869–7630	762	254
	NS5	7631–10,339	2709	903
3′-UTR	–	10,343–10,868	526	–

The immature C protein consists of mature virion C and a C-terminal hydrophobic domain. The precursor membrane protein undergoes cleavage by protease, resulting in a mature virion.

*UTR* untranslated region, *ORF* open reading frame, *C* mature virion C, *CTHD* C-terminal hydrophobic domain, *prM* precursor of membrane protein, *M* membrane protein, *E* envelope protein, *NS* non-structural protein.

**Table 2:** Identity comparison of encoded proteins between Mpulungu flavivirus and other flaviviruses.

Virus	Amino acid sequence identity to Mpulungu flavivirus (%)										
	C	PrM	E	NS1	NS2A	NS2B	NS3	NS4A	NS4B	NS5	Polyprotein
Louping ill virus	23.4	37.8	46.6	53.3	28.7	16.7	44.5	39.0	29.2	54.8	44.2
Tick borne encephalitis virus	20.9	37.4	46.2	52.1	30.5	19.8	44.7	37.3	29.6	56.2	44.5
Omsk hemorrhagic fever virus	18.0	37.1	46.6	51.5	27.8	19.0	44.2	37.0	29.6	56.6	44.2
Langat virus	22.2	40.4	44.7	50.4	23.4	17.5	43.4	37.0	30.7	55.5	43.4
Alkhurma virus	30.1	38.0	32.0	51.0	26.9	23.8	43.9	41.4	31.6	56.6	44.9
Kyasanur forest disease virus	30.1	38.0	31.6	50.7	26.1	20.6	44.0	39.8	31.2	56.5	44.6
Powassan virus	29.2	32.9	45.0	50.7	20.7	21.5	43.9	42.7	28.3	56.0	43.4
Deer tick virus	28.0	31.7	45.9	50.7	20.2	21.5	44.0	40.3	29.8	55.7	43.3
Kadam virus	21.9	35.5	46.7	47.8	24.6	27.4	41.2	34.1	27.7	55.2	42.8
Meaban virus	28.3	33.1	44.6	51.2	26.2	19.6	42.2	34.4	29.8	57.4	43.6
Saumarez Reef virus	18.0	36.8	46.3	51.5	18.9	17.4	41.4	35.4	28.2	56.4	43.0
Apoi virus	29.1	30.0	34.7	36.4	18.0	16.9	40.9	20.1	21.6	53.5	28.4
Rio Bravo virus	15.8	29.6	37.8	38.9	17.4	16.1	39.0	25.0	24.5	54.1	38.1
Zika virus	15.6	32.5	40.2	44.2	17.4	19.0	41.0	28.5	22.3	58.4	40.7
West Nile virus	< 10	30.0	38.2	41.6	19.7	23.6	40.6	28.0	24.7	59.2	40.4
Yellow fever virus	25.6	30.6	42.0	43.9	27.4	23.2	42.0	31.7	29.4	56.3	42.2
Yokose virus	21.9	35.5	39.1	42.1	20.9	24.4	38.3	28.0	26.6	54.6	39.9
Cell fusing agent virus	16.8	< 10	< 10	29.3	NA	NA	34.6	12.5	< 10	45.8	22.2
Kamiti River virus	16.6	11.1	12.9	24.5	NA	NA	31.9	< 10	< 10	46.9	25.9
Tamana bat virus	14.4	< 10	12.7	12.8	NA	NA	21.8	21.4	< 10	23.1	14.0
Ngoye virus	NA	NA	NA	NA	NA	NA	94.4*	96.8	93.3	95.8*	95.0*

CTHD and 2K protein sequences were not analyzed. *Partial amino acid sequences were used for analyses.

*NA* not analyzed.

**Figure 1:**
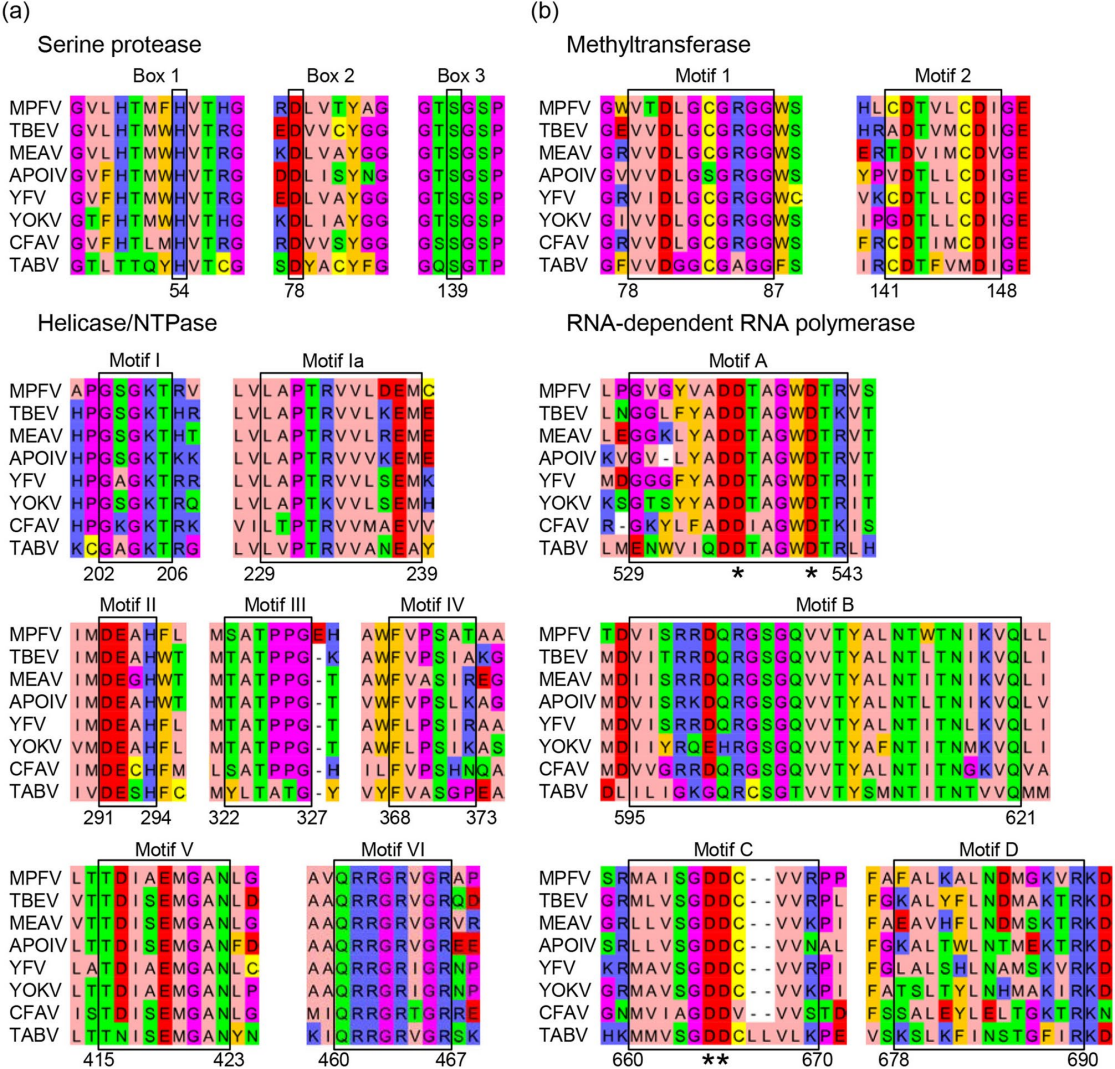
Conserved enzymatic motif in the serine protease, helicase/NTPase, methyltransferase, and RdRp. Sequence alignments of NS3 (**a**) and NS5 (**b**) from MPFV and representative flaviviruses were constructed. Enzymatic motifs are delimited accordingly. (**a**) The N-terminus and the C terminus of NS3 contain the serine protease and helicase/NTPase, respectively. The catalytic triad of serine protease (Box 1–3) and seven conserved motifs of helicase/NTPase (Motifs I–VI) are highlighted. (**b**) The N terminus and the C-terminus of NS5 contain methyltransferase and RdRp, respectively. The two (1 and 2) and four (A–D) conserved motifs of methyltransferase and RdRp are highlighted. Asterisks indicate conserved aspartic acid residues, which are important for enzymatic activity. Numbers at the bottom of the alignments refer to the MPFV sequence. Abbreviations of virus names are as follows: *MPFV* Mpulungu flavivirus, *TBEV* tick-borne encephalitis virus, *MEAV* Meaban virus, *APOIV* Apoi virus, *YFV* Yellow fever virus, *YOKV* Yokose virus, *CFAV* cell fusing agent virus, *TABV* Tamana bat virus.

NS3 and NS5 are multifunctional proteins in flaviviruses encoding enzymes required for polyprotein processing and RNA replication^31–36^. Serine protease and helicase/nucleoside-triphosphatase (NTPase) are encoded within the N- and C-terminal regions of NS3, respectively^31–33^, and NS5 contains a methyltransferase motif in the N-terminal domain as well as an RNA-dependent RNA polymerase (RdRp) in the C-terminal domain^34–36^. To analyze sequence conservation within these enzymatic motifs of NS3 and NS5, alignments were computed using MPFV sequences and those of other representative flaviviruses. The catalytic triad for serine protease activity, consisting of histidine, aspartic acid, and serine residues^31^, was conserved in the MPFV sequence at amino acid positions 54, 78, and 139, respectively (Fig. 1a). The seven sequence motifs (i.e., I, Ia, II, III, IV, V, and VI) associated with NTP hydrolysis and nucleic acid binding^32,33^, were well conserved among MPFV and other flaviviruses (Fig. 1a). The alignment of NS5 sequences indicated two (1 and 2) and four (A–D) conserved motifs related to methyltransferase and RdRp activities, respectively (Fig. 1b). The two N-terminal motifs involved in RNA cap methylation at the first nucleotide of the newly synthesized positive-strand RNA^34^ could be mapped within the MPFV sequence, and an aspartic acid residue critical for enzymatic activity^35^ was present in the MPFV NS5 sequence at amino acid position 147. Four C-terminal motifs (A–D) associated with RNA synthesis through RdRp activity were conserved among MPFV as with other flaviviruses^36^. Of these, the motifs A and C constituting the core of the catalytic site^36^ contained four important conserved aspartic acid residues in the MPFV NS5 sequence at the following positions: 536, 541, 665, and 666 (Fig. 1b). Additionally, the hydropathy profiles based on the NS3 and NS5 of MPFV were similar to those of tick-borne encephalitis virus (see Supplementary Fig. S1 online). These results suggest that the polyprotein processing and organization of its replication complex are functionally conserved among MPFV and other flaviviruses.

**Figure 2:**
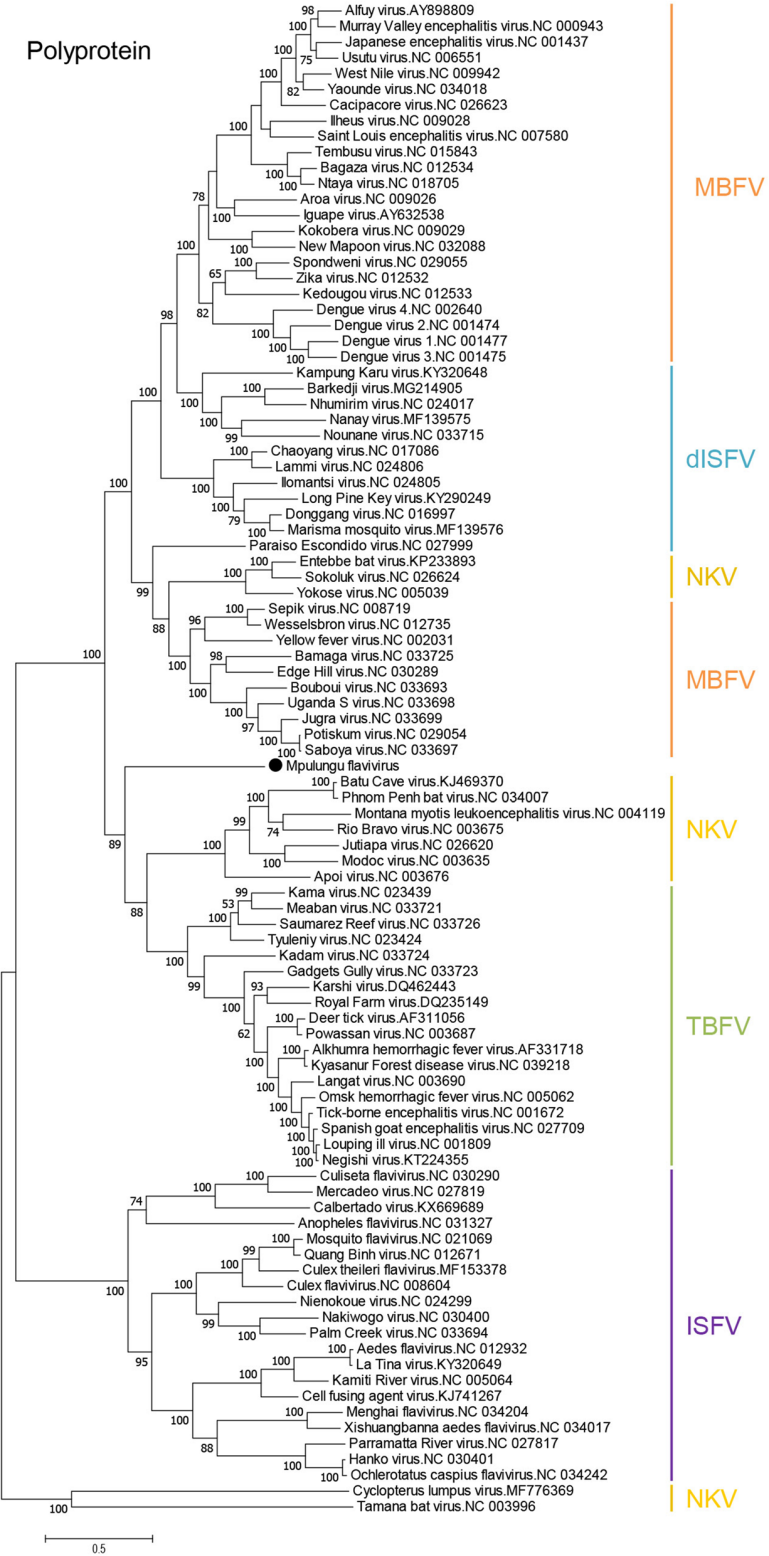
Phylogenetic analyses of flaviviruses. A phylogenetic tree based on amino acid sequences of the polyprotein was constructed using the maximum likelihood method with 1000 bootstrap replications. Bootstrap values > 50% based on 1000 replications are shown on the interior branch nodes. Scale bar 0.5 substitutions per site. Group names are indicated on the tree with abbreviations as follows: *TBFV* tick-borne flavivirus, *MBFV* mosquito-borne flavivirus, *ISFV* insect-specific flavivirus, *dISFV* dual-host affiliated insect-specific flavivirus, *NKV* no known vector. Black circle represents MPFV.

**Figure 3:**
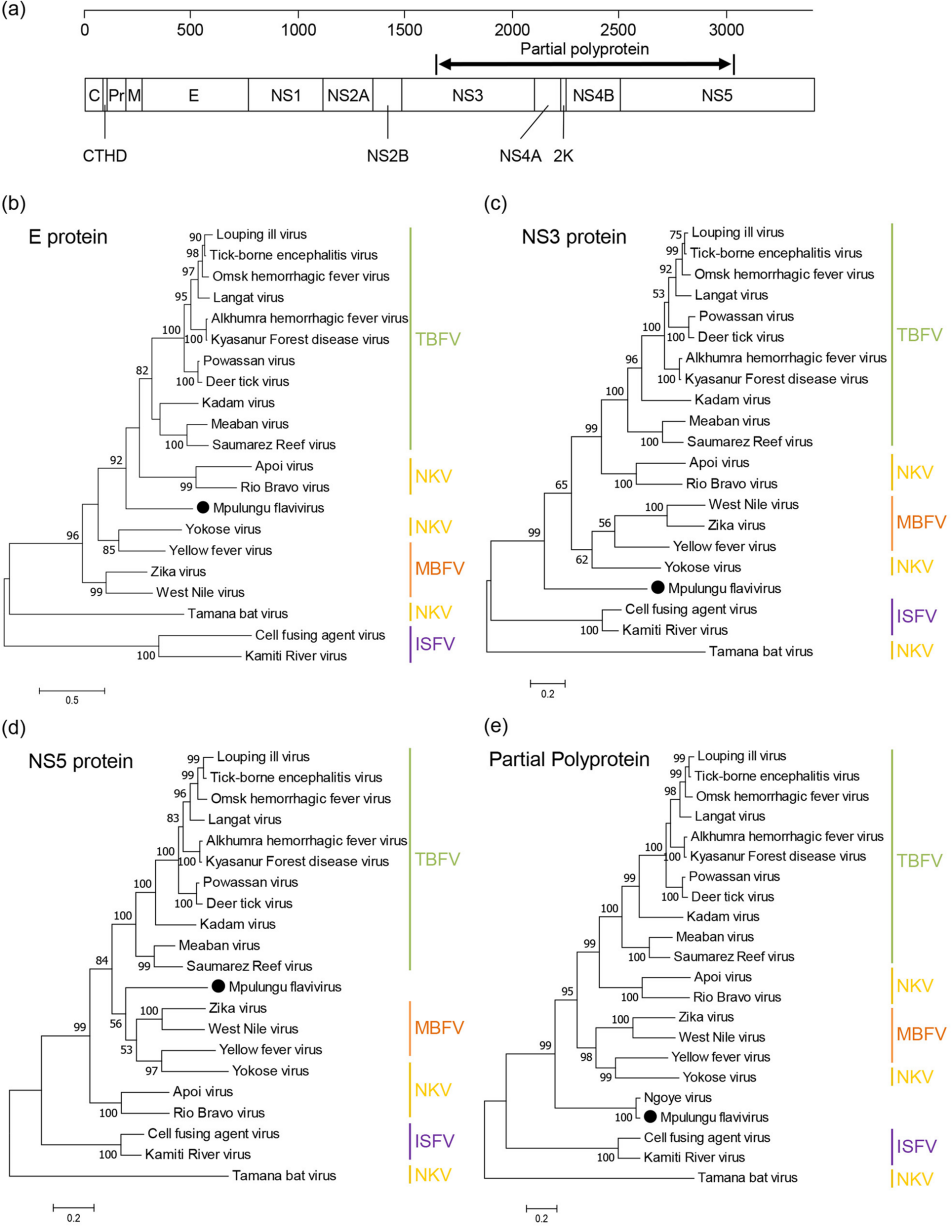
Phylogenetic analyses of E, NS3, NS5 and partial polyprotein. (**a**) Schematic diagram of MPFV polyprotein. A black double-headed arrow represents the sequenced region of Ngoye virus. Trees were constructed using the maximum-likelihood method with 1000 bootstrap replications as based upon 427–509 amino acid sequences for E (**b**), 577–623 amino acid sequences for NS3 (**c**), 855–906 amino acid sequences for NS5 (**d**), and 1392 amino acid sequences from the polyprotein corresponding to positions 1642–3033 of MPFV polyprotein (**e**). Bootstrap values > 50% based on 1000 replications are shown on the interior branch nodes, and the scale bar indicates the number of substitutions per site. Group names are indicated on the trees.

Phylogenetic analyses were conducted to investigate the evolutionary relationship among MPFV and other flaviviruses (Fig. 2). In the phylogenetic tree based on the complete consensus flavivirus polyprotein sequence (Fig. 3a), MPFV was located independently from any other known flavivirus, but shared a common origin with TBFV and TBFV-related NKV (Fig. 2). Phylogenetic analyses of E, NS3, and NS5 from MPFV and representative flaviviruses revealed that MPFV also formed a distinct branch among members of the genus *Flavivirus* (Fig. 3b–d). These results suggest that MPFV is the first known representation of a flavivirus that shares ancestral roots with TBFVs and TBFV-related NKVs. To clarify the phylogenetic relationship between MPFV and Ngoye virus, we constructed a phylogenetic tree based on 1392 amino acid sequences from the partially identified polyprotein of Ngoye virus (Fig. 3a). MPFV clustered only with Ngoye virus, with a unique lineage being formed by MPFV and Ngoye virus (Fig. 3e). These results indicate that tick-related flaviviruses from ticks collected in Africa (i.e., Senegal and Zambia) may form a distinct flavivirus group.

### Analysis of the dinucleotide composition of MPFV

**Figure 4:**
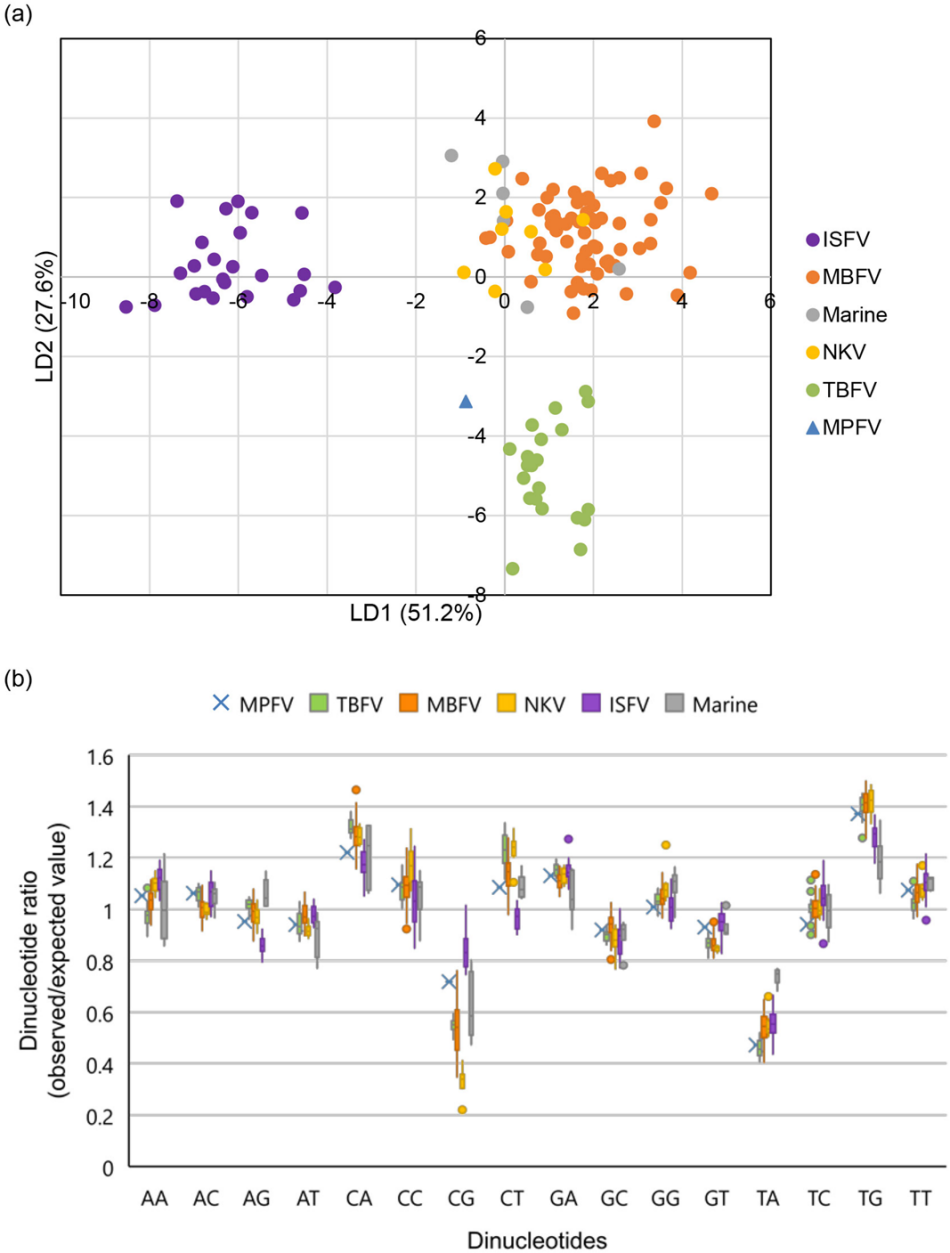
Nucleotide composition analysis of flaviviruses. (**a**) Score plot of the linear discriminant analysis (LDA). The figure shows a scatterplot of the two discriminant scores explaining the largest amount of the components from LDA (51.2% and 27.6% for LD1 and LD2, respectively). (**b**) Boxplot of dinucleotide ratios in flavivirus groups. Blue triangle (**a**) and blue cross (**b**) represent MPFV. Green: TBFV, orange: MBFV, yellow: NKV, purple: ISFV, gray: Marine.

Analysis of nucleotide and dinucleotide contents of viruses and their hosts indicates that flaviviruses have coevolved with the latter^37,38^. To predict the host range of MPFV based on dinucleotide usage, a linear discriminant analysis was performed based on the dinucleotide ratios in complete or nearly complete genome sequences from 128 flaviviruses with a defined host range and transmissibility, which has been previously reported to determine host specificity (Fig. 4a)^38–41^. MPFV was predicted as a TBFV based on five flavivirus groups via this analysis.

In addition, we compared dinucleotide ratios for 16 dinucleotides across MPFV and five flavivirus groups (Fig. 4b). As a threshold of dinucleotide patterns, Karlin and Mrazek showed that dinucleotide ratios < 0.78 can be regarded as underrepresented dinucleotides, whereas values > 1.23 indicate overrepresented dinucleotides^42^. All vertebrate-infecting flavivirus groups (i.e., NKV, MBFV, and TBFV) contain underrepresented CpG and TpA, and overrepresented TpG and CpA. These characteristic dinucleotide ratios are commonly found in vertebrate-infecting viruses and mimic the vertebrate genome signature^37,38^. MPFV also exhibits these common characteristics, indicating that the virus may belong to TBFV, vertebrate-infecting flavivirus groups.

### Characterization of RNA secondary structures of MPFV in the UTRs

**Figure 5:**
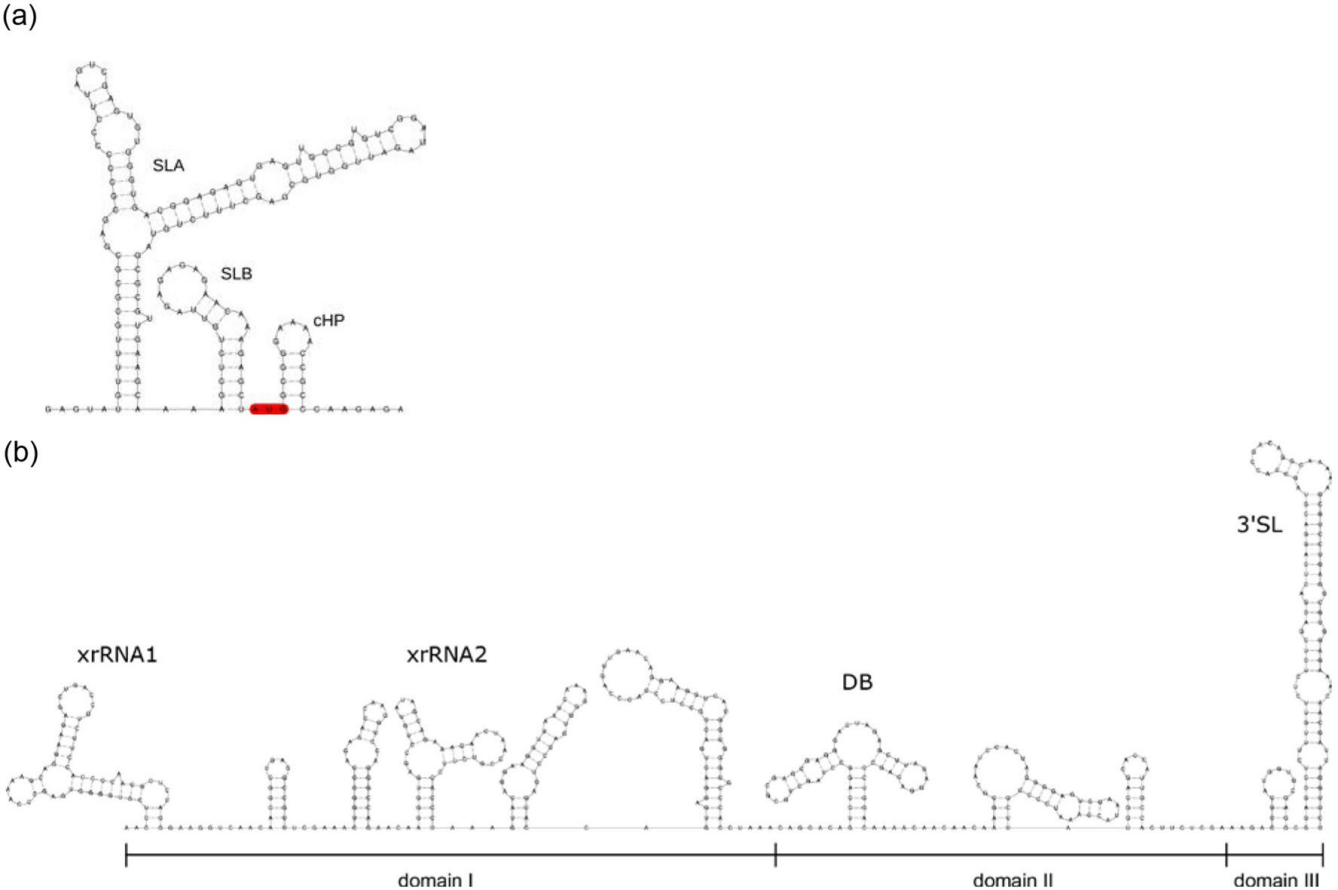
Predicted secondary structures of the 5′- and the 3′-UTRs of MPFV. (**a**) Secondary structure prediction of the 5′-terminus was performed using the nucleotide sequence of the 5′-UTR and the adjacent portion of the capsid protein. The canonical start codon is highlighted in red. The 5′-UTR of MPFV contains evolutionarily conserved elements, namely stem-loops A (SLA) and B (SLB), followed by the conserved capsid hairpin (cHP) structure at the beginning of the coding regions. (**b**) Secondary structure prediction of the 3′-UTR shows the overall architecture of this regulatory region, containing two exoribonuclease-resistant RNAs (xrRNA1 and xrRNA2), a dumbbell (DB) element and a long terminal 3′-stem-loop (3′SL) structure. For the other stem-loop structures, no evolutionary support among other flaviviruses was obtained.

In addition to identifying characteristic evolutionary patterns in the coding regions, we performed in silico thermodynamic modelling of the MPFV UTRs. Both UTRs are known to harbor unique RNA structural elements that are evolutionarily conserved among many flaviviruses^30^. The 5′-UTR of MPFV folded into canonical stem-loop A (SLA) and B (SLB) structures, and is immediately followed by a capsid hairpin element (cHP; Fig. 5a). The overall composition of the 5′-UTR resembles that of other flaviviruses^43,44^ with an extended SLA side-stem loop characteristic of TBFVs (unpublished data). Similarly, the MPFV 3′-UTR consists of a set of functional elements that are organized into three domains and exhibit structural homology to known elements within other flaviviruses. These included two distinct xrRNA structures, a dumbbell (DB) element as well as a conserved terminal 3′ stem-loop (3′SL) structure (Fig. 5b). Other stem-loops predicted in the MPFV 3′-UTR did not show homology to known functional elements. The MPFV DB element is of particular interest, given that DB elements were typically not found in the 3′-UTRs of mammalian or seabird-associated TBFVs, but were present in several TBFV-related NKVs^30^, with which MPFV shared ancestral roots. Although the proximal stem-loop originating from the central multi-loop of the MPFV DB element was considerably shorter than the DB elements of MBFVs or dual-host affiliated ISFVs^30,45^, for example, MPFV was unique among the known TBFVs to contain such an element in its 3′-UTR.

### In vitro analysis of MPFV xrRNA structures for exoribonuclease-resistance

**Figure 6:**
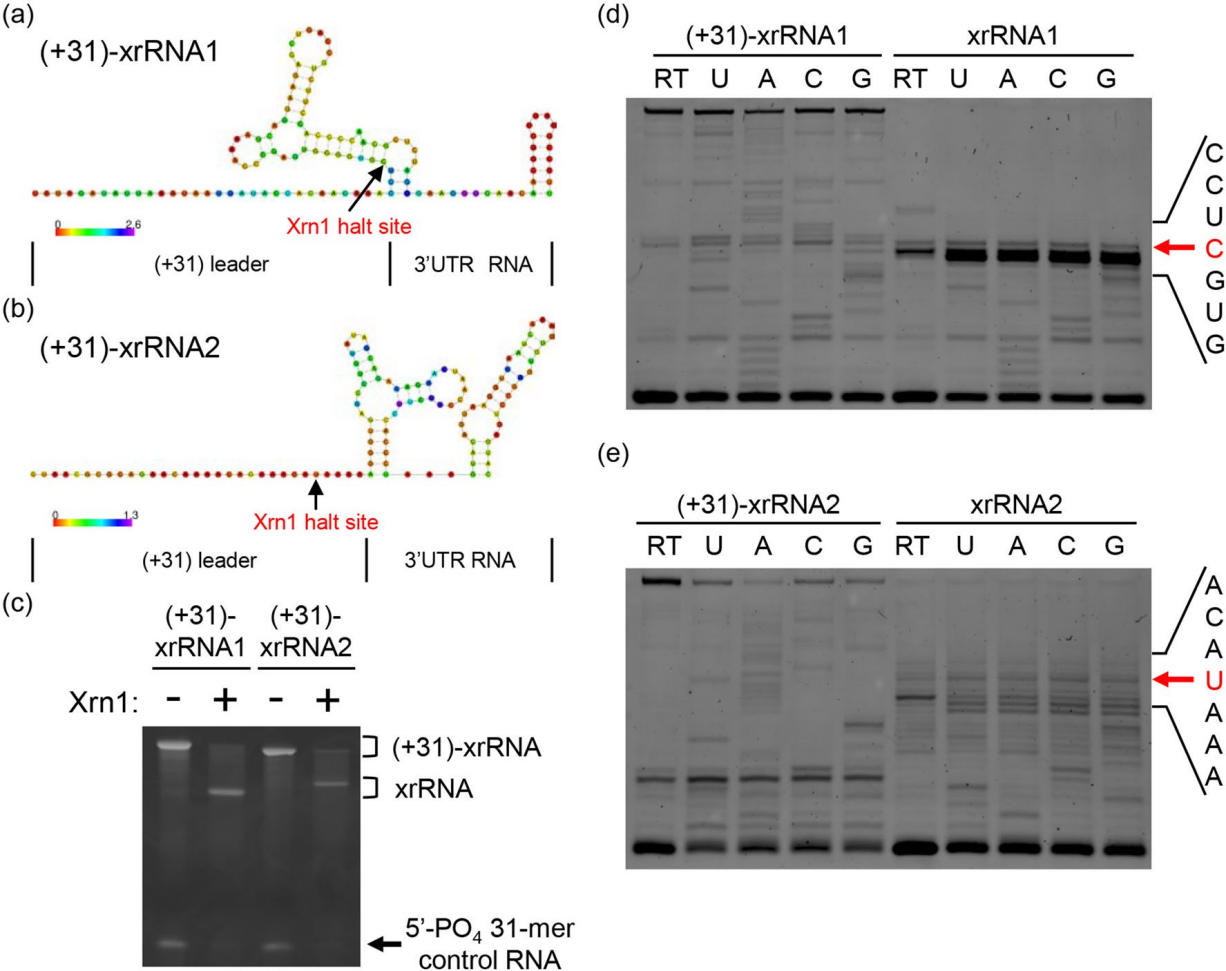
Xrn1 degradation assay and characterization of the Xrn1-resistant products. (**a**,**b**) Schematic diagram of predicted secondary structures of two RNA constructs, (+ 31)-xrRNA1 (**a**) and (+ 31)-xrRNA2 (**b**), used for the in vitro Xrn1 degradation assay. The leader sequences in these constructs are artificially designed 31-mers which do not form a prominent structure nor interact with the downstream genomic sequences of xrRNA structures. Heat scale bars represent bits of positional entropy. Black arrows indicate the Xrn1 halt sites. (**c**) In vitro Xrn1 degradation assay using (+31)-xrRNA1 and (+31)-xrRNA2. This image is a part of full-length gel represented in Supplementary Fig. S3a. (**d**,**e**) Reverse transcription mapping the Xrn1 halt sites with RNA from panel (**c**). The location of the stop site (the 5′ border of the RNA products) is shown with a red arrow to the right, along with the sequence of the RNA surrounding this position. These images are parts of full-length gels represented in Supplementary Fig. S3b,c.

Exoribonuclease-resistant RNAs are short RNAs, typically 60–90 nucleotides in length, with the capacity to stall exoribonucleases. They are commonly comprised of a three-way junction element, downstream hairpin-loop and transient RNA pseudoknot^15,30^. The xrRNA structures located in flaviviral 3′-UTRs contribute to the production of sfRNAs formed by partial degradation of viral RNA via cellular 5′–3′ exoribonucleases such as Xrn1. Although the RNA structures observed in the 3′-UTR of MPFV were structurally homologous to previously described xrRNA structures^23,24^, a covariance model analysis against known xrRNA structures of other known flaviviruses did not yield any plausible hits. This is likely due to the fact that MPFV xrRNA structures are sufficiently diverged from the xrRNA structures of previously described flaviviruses that have been used to construct covariance models. To confirm whether these RNA elements can stall exoribonucleases, Xrn1 degradation assays were performed in vitro. Through an RNA design approach based on RNAblueprint^46^, we designed two RNA constructs, consisting of potential xrRNA-forming sequences of MPFV and 31-nucleotide leader sequences that do not interact with the xrRNA three-way junction fold. The reliability of the predicted structures was assessed in terms of positional entropy, highlighting that the leader sequence is effectively unpaired (Fig. 6a,b). The Xrn1 degradation assays using these two RNA constructs [(+31)-xrRNA1 and (+31)-xrRNA2] revealed that xrRNA1 and xrRNA2 effectively blocked the progression of Xrn1 (Fig. 6c). The precise locations at which Xrn1 is blocked by xrRNA structures were mapped using a primer extension method. The Xrn1 halt site of partially degraded xrRNA1 was located in the basal stem of xrRNA1 (Fig. 6a,d). Conversely, xrRNA2 analysis revealed that the truncated RNA ends with an uracil, 5 nucleotides upstream of the three-way junction secondary structure (Fig. 6b,e). While the halt site in the xrRNA1 construct is unexpectedly located in a stem-loop structure, we attribute this phenomenon to the high conformational flexibility of the basal 3-nucleotide stem in the predicted (31)-xrRNA1 structure, as expressed by high positional entropy (Fig. 6a). On the contrary, the entire closing stem of (31)-xrRNA2 exhibited low positional entropy, indicating that this predicted structure was well defined (Fig. 6b). These results suggest that MPFV forms two unique functional xrRNA structures in the 3′-UTR, and that the sfRNA of MPFV can be produced as observed in other flaviviruses.

## Discussion

In this study, we describe the discovery of a novel flavivirus tentatively named MPFV, detected from a *Rhipicephalus muhsamae* in Zambia and have determined its complete genome sequence including the 5′-UTR, the ORF, and the 3′-UTR. MPFV was found to be closely related to the Ngoye virus previously detected in Senegal^12^, and these flaviviruses were phylogenetically distinct from other TBFVs. While TBFVs have been mainly detected in Europe, North America, Siberia, and Far East regions^47^, our results suggest that a novel TBFV group, including MPFV and Ngoye virus, may exist on the African continent. Considering the long distance (> 5000 km) between Zambia and Senegal, the discovery of MPFV and Ngoye virus suggests that TBFVs belonging to a novel TBFV group may be widely distributed in Africa. Our findings provide new information regarding the geographical distribution and genetic diversity of flaviviruses.

Unfortunately, the isolation of MPFV using cell culture and neonatal mice was unsuccessful in the present study. In a previous report, newborn mice and several cell lines were used for isolation of Ngoye virus, but viral amplification was similarly not observed as seen in our studies^12^. Thus, we predicted the host range of MPFV using a linear discriminant analysis based on the dinucleotide ratios. Flaviviruses mimic the dinucleotide composition of their hosts, indicating that there is a clear difference in dinucleotide composition between vertebrate-infecting and invertebrate-specific flaviviruses^37^. A previous study showed linear discriminant analysis for Flaviviridae with a high sensitivity rate, as 99% of vector-borne flaviviruses were accurately predicted within the current flavivirus group^38^. Therefore, dinucleotide compositions were thought to be a reliable tool for predicting the host range of flaviviruses. Our compositional analyses showed that MPFV was predicted to be a TBFV and exhibited similar dinucleotide composition characteristics with vertebrate-infecting flaviviruses. These results suggested that MPFV might infect vertebrate hosts in the same manner as other TBFVs, which could potentially impose a public health risk. Ngoye virus was detected in ticks captured from ovine and caprine species, and it would be of interest to investigate the genomic detection and the serosurveillance of MPFV in livestock. Recently, novel flaviviruses were detected in marine vertebrates and invertebrates^48–50^, and the genome of crustacean flaviviruses—namely marine invertebrate flaviviruses—were shown to be related to terrestrial vector-borne flaviviruses^48^. Thus, improved understanding of the potential origins of invertebrate-vertebrate flaviviruses is likely necessary to explore potential vertebrate hosts of MPFV.

Both the 5′- and the 3′-UTRs of flaviviruses contain sequence motifs related to viral translation and replication^16–22^. The secondary structure and function of the 5′-UTR and areas adjacent to the capsid coding region are well conserved among flaviviruses and contain three RNA elements with crucial functions (SLA, SLB, and cHP)^16–21^. The large SLA positioned in the 5′-terminus of flaviviral genomes plays a role as a promoter element recognized by viral RdRp; binding of RdRp to SLA is necessary for viral RNA synthesis^16,17^. The second short SLB, often containing an initiation AUG codon, is complementary to a sequence present at the 3′-end of the viral genome, and is also important for viral replication^18,19^. The cHP in the C coding region enhances recognition of the AUG codon, and is required for RNA synthesis and late translation of viral proteins^20,21^. Our data indicate that the MPFV genome contains a typical flavivirus 5′-UTR structure, including canonical SLA, SLB, and cHP structures. Thus, MPFV viral RNA synthesis would be expected to be conducted in a similar manner as other flaviviruses. Conversely, the organization of the 3′-UTR differs between flaviviruses, and sequence composition, length, and secondary structures are also varied considerably^30^. Nevertheless, some RNA secondary structures are shared among the flavivirus groups^30^. Of these, the terminal 3′SL structures, which are associated with long-range RNA-RNA interactions between the 5′- and the 3′-UTRs, are required for virus replication, and are therefore present in the 3′ termini of all flaviviruses^17^. Although MPFV contained similar 3′SL structures to other TBFVs, other secondary structures differed from those of known TBFVs. DB elements, for example, are not conserved within TBFVs, but are conserved between MBFVs and dual-host affiliated ISFV. Although the proximal stem-loop originating from the central multi-loop of the DB element in the 3′-UTR of MPFV and TBFV-related NKVs is shorter than those of MBFVs and dual-host affiliated ISFVs, the biological and functional significance of these differences remains unclear. Generally, the 3′-UTRs of most MBFVs have two DB elements, which are related to genomic cyclization and optimal translation^22^. Further research on genomic cyclization and translation may be required for the accurate biological characterization of the shorter stem-loops within DB elements in MPFV and TBFV-related NKVs. For other MPFV stem-loop structures, no evolutionary support was obtained among other flaviviruses following a bioinformatic homology search. These results indicate that the 3′-UTR of MPFV is unique and may help to the understanding of the evolutionary history of the 3′-UTR in flaviviruses.

We demonstrated through in vitro exoribonuclease assays that two xrRNA structures derived from the 3′-UTR of MPFV achieved Xrn1 resistance. X-ray crystallography of xrRNA structures derived from MBFVs revealed a three-way junction and a pseudoknot interaction creating an unusual and complex fold, and forming a ring-like structure during folding^51,52^. The 5′ end of the xrRNA structure passes through the center of the ring-like structure, and this feature results in Xrn1 resistance to xrRNA structures. A pseudoknot interaction, which might be transient, is formed via base pairing (2–7 nucleotides) between the apical xrRNA loop and downstream sequence^24^. Within the xrRNA1 of MPFV, putative base pairing occurs between a 5′-UGACC-3′ sequence in the apical loop and a 5′-GGUCA-3′ sequence 26 nucleotides downstream; the complementary sequence of the apical loop also appears within a downstream stem-loop structure for xrRNA2 (see Supplementary Fig. S2 online). These putative pairing sequences might be related to a tertiary interaction during formation of these xrRNA structures. Since these predictions are merely suggested interactions and without the support of thermodynamic-based modeling, further studies of RNA structures using X-ray crystallography are required to clarify the mechanism of exoribonuclease resistance for xrRNA structures.

In general, multiple xrRNA structures are present in the 3′-UTR of flaviviruses, producing multiple sfRNAs^25^. Although the pattern of sfRNA production in human cells infected with West Nile virus demonstrated that the longest sfRNA was efficiently produced by the first xrRNA structure, the second xrRNA structure containing certain mutations and abrogating tertiary interactions also led to a decrease in the amount of the longest sfRNA, suggesting that xrRNA duplication in the 5′ end of the 3′-UTR is required for efficient production of sfRNA via an unknown mechanism^25^. The two functional MPFV xrRNA structures were located in succession near the 5′ end of the 3′-UTR and might contribute to the production of complete sfRNA in MPFV due to incomplete degradation of RNA by the host exoribonuclease. Duplications of xrRNA structures at the 5′ end of the 3′-UTR are common in vector-borne flaviviruses, such as MBFV and TBFV, and were uncommon in the 3′-UTRs of ISFV^29^. The xrRNA duplication observed in vector-borne flaviviruses is related to host adaptation without reduction of viral fitness during host switching between invertebrates and vertebrates^25,29^. The first xrRNA structure of West Nile virus, when mutated to disrupt its structure, significantly led to a reduction in its pathogenicity, indicating that this xrRNA structure plays a role in virulence to vertebrate hosts^28^. Interestingly, deletion of the second xrRNA structure in Dengue virus led to an increase in viral replication capacity in mosquito cells, whereas deletion of both xrRNA structures reduced viral replication capacity in mammalian cells^29^. In previous studies, a new model was proposed in which vector-borne flaviviruses containing xrRNA structures duplication maintain the potential for efficient sfRNA production by maintaining the intact first xrRNA structure in both vertebrates and invertebrates, but in invertebrates these viruses modulate the second xrRNA structure to enable adaptation to their host^25,29^. Therefore, it is reasonable to assume that MPFV is a vector-borne flavivirus containing duplicated xrRNA structures, and further exploration is needed to assess its significance.

## Methods

### Detection of flaviviruses

Tick RNA samples were examined to detect flavivirus via RT-PCR using a One Step RT-PCR Kit v2 (Takara, Shiga, Japan) with pan-flavivirus primer set (see Supplementary Table S2 online) based on the conserved sequence within the flavivirus NS5 protein as previously described^54^. The RT-PCR conditions were as follows: initial reverse transcription step at 50 °C for 30 min; PCR activation step at 94 °C for 2 min; 43 cycles of 94 °C for 30 s, 53 °C for 30 s, and 72 °C for 30 s; and a final extension at 72 °C for 5 min. PCR products were subjected to direct sequencing using the Big Dye Terminator v3.1 Cycle Sequencing Kit (Applied Biosystems, Foster City, CA).

### Virus isolation

Tick homogenates positive for flavivirus were cultured in Vero E6 (kindly provided by Dr. Heinz Feldmann, National Institutes of Health, Bethesda, MD), BHK-21 (gift from Dr. Akira Oya, the National Institutes of Health, now the National Institute of Infectious Diseases, Tokyo, Japan), ISE6 (kindly provided by Dr. Ulrike Munderloh, University of Minnesota, Saint Paul, MN) or C6/36 cells (purchased from the American Type Culture Collection, Manassas, VA). Tick lysates were inoculated into these cell lines, and supernatants and cell lysates were examined at each passage for detection of flavivirus genome by RT-PCR of the NS5 region. Lysates were also inoculated into neonatal mice brain via intracerebral injections, and brain-derived RNA was subsequently subjected to RT-PCR. All experiments for virus isolation were performed at the Biosafety Level-3 facility at the Research Center for Zoonosis Control, Hokkaido University following the institutional guidelines.

### Library preparation and whole genome sequencing

Total RNAs extracted from tick homogenates positive for flavivirus were used for whole genome sequencing. Ribosomal RNA depletion from total RNA was performed using RiboMinus Eukaryote Kit for RNA-Seq (Invitrogen), and cDNA was synthesized using a PrimeScript Double Strand cDNA Synthesis Kit (Takara) according to the manufacturers’ instructions. The cDNA libraries were prepared using a Nextera XT DNA Library Preparation Kit (Illumina, San Diego, CA) according to the manufacturer’s instructions, and were then subjected to whole-genome sequencing on a MiSeq using a MiSeq Reagent Kit v3 (600 cycles) (Illumina). Sequencing data was analyzed using the CLC Genomics Workbench 12.0 (https://digitalinsights.qiagen.com; CLC bio, Hilden, Germany). Flavivirus genome contigs were obtained by de novo assembly and the overlapped contig sequences were confirmed by PCR amplification with specific primers and Sanger sequencing. The 5′ and 3′ termini of the flavivirus genome were amplified using RACE with specific primers and a SMARTer RACE cDNA Amplification Kit (Takara) according to the manufacturer’s protocol (see Supplementary Table S2 online). Amplified products were directly sequenced using a BigDye Terminator v3.1 Cycle Sequencing Kit.

### Genetic comparison and phylogenetic analyses of flaviviruses

Polyprotein ORF positions were predicted using GENETYX version 12 (https://www.genetyx.co.jp/; GENETYX Corporation, Tokyo, Japan). The putative cleavage sites of the detected flavivirus were determined by comparison with known cleavage sites of previously characterized flaviviruses as well as cleavage patterns of a host signal peptidase, furin and viral serine protease as previously described^55^. Bioinformatic analyses were performed using flavivirus sequences deposited in the DDBJ/EMBL-Bank/GenBank databases. Identity comparison analyses were conducted among flaviviruses using GENETYX version 12. Conserved enzymatic motifs of NS3 and NS5 were identified by sequence alignments of detected and previously characterized flaviviruses. The hydropathy profiles of viral proteins were obtained using the web-based tool ProtScale (https://web.expasy.org/protscale/) from the ExPASy Bioinformatics Resource Portal with the Kyte and Doolittle scale option^56^. Phylogenetic analyses based on the amino acid sequence of flavivirus polyprotein and each viral protein were performed using MEGA7^57^. The MUSCLE protocol was used to align the sequences, and phylogenetic trees were constructed using the maximum-likelihood method based on the Tamura-Nei model with 1000 bootstrap replicates.

### Compositional analysis

Complete or nearly complete genome sequences from 128 flaviviruses with defined host range and transmissibility were used as a dataset, which were classified as TBFV, MBFV, NKV, ISFV, and flaviviruses derived from marine organisms (i.e., Marine), and used for analyses (see Supplementary Table S4 online). The dinucleotide ratios (observed/expected values) were calculated using the formula P_XY_ = ƒ_XY_/ƒ_X_ƒ_Y_, in which ƒ_X_ and ƒ_Y_ denote the frequencies of the mononucleotides X and Y, respectively, and ƒ_XY_ denotes the frequency of dinucleotide XY^42^. Linear discriminant analysis was performed using the R package (https://www.R-project.org)^58^.

### Prediction of RNA secondary structure

RNA secondary structure predictions in both MPFV UTRs were computed using RNAfold of the Vienna RNA Package 2.0 (https://www.tbi.univie.ac.at/RNA/)^59^, explicitly disallowing isolated base pairs (–noLP option). Structural homology of the predicted 5′-UTR stem-loop structures SLA and SLB to these elements in other flaviviruses was determined by Infernal covariance models (CMs)^60^. Likewise, TBFV-specific CMs from a recent study^30^ were used to confirm the predicted locus of the MPLV 3′SL element, and the Rfam^61^ CM RF00525 (Flavivirus_DB) was used to annotate the single DB element in the 3′-UTR. All secondary structure plots were produced with the RNAplot utility^59^.

### Design of leader sequences for the Xrn1 degradation assay

To test the capacity of the predicted 3′-UTR structures xrRNA1 and xrRNA2 to inhibit nuclease digestion, we performed an Xrn1 degradation assay according to the methods of Chapman et al.^23^. To this end, we extracted the nucleotide sequences folding into three-way junction structures together with their downstream hairpins. The degradation assay requires a leader sequence upstream of the xrRNA structure to load Xrn1. To exclude the possibility that the leader sequence interacts with the sequence forming the three-way junction, we designed custom, artificial 31 nt leader sequences that do not form any significant secondary structures nor distort the canonical xrRNA fold. We sampled sequences fulfilling this requirement using RNAblueprint^46^. We employed partition function folding with default parameters implemented in the ViennaRNA package to obtain ensemble free energies ΔG, optimizing for a maximal (Z^F^/Z) ratio as design goal, where Z^F^ is the partition function under the constraint that the three-way junction and downstream hairpin are formed in the presence of the designed 31 nt leader sequence, and Z is the unconstrained partition function. Z is related to the ensemble free energy via ΔG = − RTlnZ, where R is the universal gas constant and T is the thermodynamic temperature. Reliability of the predicted structures, including the designed leader sequences was, visualized in terms of positional entropy (Fig. 6a,b).

### Xrn1 degradation assay

Two 3′-UTR RNAs [(+ 31)-xrRNA1 and (+ 31)-xrRNA2] were chemically synthesized and purified by the Agilent 1290 Infinity II chromatography system (Agilent Technologies, Santa Clara, CA; see Supplementary Table S5 online). The synthesized RNAs were phosphorylated using T4 Polynucleotide Kinase (Takara) and purified by the RNeasy MinElute Cleanup Kit (Qiagen, Hilden, Germany) according to the manufacturer’s instructions. The modified RNAs were incubated at 90 °C for 2 min, followed by 20 °C for 5 min, then held at 4 °C for RNA folding. Xrn1 digestion reactions were conducted with 3–4 µg of the 3′-UTR RNA (~ 100 pmol) and 2 µg of 5′ monophosphorylated 31-mer control RNA (~ 200 pmol) in NEB3 buffer [100 mM NaCl, 50 mM Tris–HCl (pH 7.9), 10 mM MgCl_2_, and 1 mM DTT] (New England Biolabs, Ipswich, MA). The RNA mixture was split between two tubes, and two units of Xrn1 (New England Biolabs) were added to one aliquot while the other served as an Xrn1-negative control. Both mixtures were incubated at 37 °C for 2 h, followed by quenching via addition of an equal volume of Novex TBE-Urea Sample Buffer (Invitrogen). The RNA products were analyzed on 15% denaturing PAGE gel and visualized by ethidium bromide staining.

### Mapping Xrn1 halt sites of 3′-UTR RNAs

Xrn1 halt sites were mapped by RNA sequencing utilizing the primer extension method^62^. RNA products remaining after the Xrn1 degradation assay were recovered using the ZR small-RNA PAGE Recovery Kit (ZYMO research, Irvine, CA) according to the manufacturer’s instruction. Reverse transcription was carried out using SuperScript IV Reverse Transcriptase (Invitrogen) according to the manufacturer’s instruction. Briefly, approximately 2 pmol of recovered RNA products were annealed to the 5′ end-labeled primer with deoxyribonucleoside triphosphates and dideoxynucleoside triphosphates (see Supplementary Table S2 online). Reverse transcriptase and buffer components were added to the primer-RNA hybrids to catalyze elongation of the primer to 5′ end of the RNA. Following the elongation reaction, equal volumes of loading buffer (95% formamide and 10 mM EDTA) were added to the reactions, and the mixtures were then incubated at 80 °C for 5 min. The resulting fluorescein-labeled cDNA products were analyzed on a 15% denaturing PAGE gel and visualized by the ChemiDoc Touch Imaging System (Bio-Rad Laboratories, Inc., Hercules, CA).

### Ethical statement and sample collection

All animal experiments were performed with approval from the Animal Care and Use Committee of Hokkaido University following the Fundamental Guidelines for Proper Conduct of Animal Experiment and Related Activities in Academic Research Institutions under the jurisdiction of the Ministry of Education, Culture, Sports, Science and Technology in Japan (permit number 19-0019). This study was carried out in compliance with the ARRIVE guidelines for experiments involving animals^53^. Adult host-questing ticks were captured using the flagging method, and blood-sucking ticks were collected from domestic animals (e.g., cattle, goat, sheep, and dogs) by permissions of the owners obtained by the veterinarians in Isoka (10.15 °S, 32.63 °E), Mpulungu (8.76 °S, 31.11 °E), and Samfya (11.36 °S, 29.55 °E) from November 2017 to January 2018 in Zambia. Collected ticks (n = 573) were morphologically identified under a stereomicroscope (see Supplementary Table S1 online). Each tick was washed in 70% ethanol containing 1% iodine and then submerged in distilled water. The samples were then homogenized with 200 μl of Dulbecco’s modified Eagle’s medium using a homogenizer (Tomy Seiko, Tokyo, Japan) at 3000 rpm. Total RNAs were extracted from 100 μl of the homogenates using TRIzol-LS (Invitrogen, Waltham, MA) according to the manufacturer’s protocol, and remaining lysate samples were stored at − 80 °C until use for virus isolation.

## Supplementary Information

Supplementary Information

## Data Availability

The sequence of Mpulungu flavivirus was deposited into the GenBank/EMBL/DDBJ database (Accession No. LC582740). All data generated or analyzed within this study are included in this published manuscript and its supplementary information files.
