# Supplementary material for: An African tick flavivirus forming an independent clade exhibits unique exoribonuclease-resistant RNA structures in the genomic 3′-untranslated region
Source: Sci Rep. 2021 Mar 1;11:4883. doi: 10.1038/s41598-021-84365-9 (PMC7921595; doi:10.1038/s41598-021-84365-9)
Supplement: Supplementary file 1 — Supplementary Information [file 41598_2021_84365_MOESM1_ESM.pdf]

Scientific Reports

Research Articles

An African tick flavivirus forming an independent clade exhibits unique exoribonuclease-resistant RNA structures in the genomic 3′-untranslated region

Hayato Harima, Yasuko Orba, Shiho Torii, Yongjin Qiu, Masahiro Kajihara, Yoshiki Eto, Naoya Matsuta, Bernard M. Hang'ombe, Yuki Eshita, Kentaro Uemura, Keita Matsuno, Michihito Sasaki, Kentaro Yoshii, Ryo Nakao, William W. Hall, Ayato Takada, Takashi Abe, Michael T. Wolfinger, Martin Simuunza, Hirofumi Sawa*

*Corresponding author: Hirofumi Sawa

Division of Molecular Pathobiology, Research Center for Zoonosis Control, Hokkaido University, Japan

E-mail: h-sawa@czc.hokudai.ac.jp

Supplementary Table S1. Summary of ticks captured in Zambia

Location	Date	Tick species	Sex	Number of ticks
Isoka	November, 2017	*Amblyomma variegatum*	Male	35
			Female	25
		Argas walkarea	NI	20
		Rhipicephalus (*Boophilus*) *microplus*	Male	5
			Female	41
		Rhipicephalus (*Boophilus*) sp.	Female	1
		Haemaphysalis leachi	Female	1
		Rhipicephalus lunulatus	Male	39
			Female	94
		Rhipicephalus sanguennius	Male	6
			Female	6
		Rhipicephalus zambeziensis	Male	26
			Female	21
		Rhipicephalus sp.	Male	1
			Female	1
Mpulungu	November, 2017	*Amblyomma variegatum*	Male	23
			Female	19
		Amblyomma pomposum	Male	3
			Female	2
		Rhipicephalus (*Boophilus*) *microplus*	Female	5
		Rhipicephalus (*Boophilus*) sp.	Female	7
		Haemaphysalis leachi	Male	6
			Female	3
		Rhipicephalus appendiculatus	Male	9
			Female	3
		Rhipicephalus camicasi	Male	1
		Rhipicephalus compositus	Female	1
		Rhipicephalus evertsi evertsi	Female	1
		Rhipicephalus guilhoni	Male	2
		Rhipicephalus lunulatus	Male	2
			Female	8
		Rhipicephalus muhsamae	Male	2
		Rhipicephalus sanguennius	Male	26
			Female	10
		Rhipicephalus simpsoni	Male	1
		Rhipicephalus sulcatus	Male	4
			Female	2
		Rhipicephalus zambeziensis	Male	5
			Female	2
		Rhipicephalus sp.	Male	14
			Female	19
Samfya	January, 2018	*Amblyomma variegatum*	Male	13
			Female	23

	Rhipicephalus (*Boophilus*) *microplus*	Male	4
		Female	30
	Rhipicephalus appendiculatus	Female	1
	Total		573

NI; Not identified

Supplementary Table S2. Primers used in this study

Primer	Sequence (5'-3')	Reference
Flavi all S	TACAACATgATggggAARAgAgARAA	
DEN4	TACAACATgATgggRAAACgTgAGAA	[1]
Flavi all AS2	gTgTCCCAgCCNgCKgTgTCATCWgC	
MPFV-5RACE-R	gattacgccaagcttGAACACATGTGCTCCGAGGGCTAG	
MPFV-5RACE-nested-R2	gattacgccaagcttCTGGCCTGTATTTGCTCTCCGTCTC	
MPFV-3RACE-F	gattacgccaagcttCAAAGCGCAATCCACTAGGGGAGTG	This study
5’FAM-xrRNA1-R	[FAM]-AGACGGTCAGCTCTTCCTGC	
5’FAM-xrRNA2-R	[FAM]-AGACGGTTAGTTCTTTCTGC	

Supplementary Table S3. Putative polyprotein cleavage sites of Mpulungu flavivirus and other representative flaviviruses

Virus	Cleavage site (protease)					
	C/CTHD (VSP)	AnchC/prM (Signalase)	pr/M (Furin)	M/E (Signalase)	E/NS1 (Signalase)	NS1/NS2A (Unknown)
Mpulungu flavivirus	AGRRR / ANTGQ	SLALG / AHVFM	RIAER / SLSVT	APAYG / TQCLG	LGVGA / DVACG	GFVYA / WALEV
Ngoye virus	-	-	-	-	-	-
Louping ill virus	RGKRR / SVTNW	GMTLA / ATVRK	SRTRR / SVLIP	APVYA / SRCTH	LGVGA / DVGCA	SMVVA / DNGEL
Tick borne encephalitis virus	RGKRR / SATDW	GMTLA / ATVRK	SRTRR / SVLIP	APVYA / SRCTH	LGVGA / DVGCA	SMVVA / DNGEL
Omsk hemorrhagic fever virus	RGKRR / STTDW	SIALA / ATVRK	TRSRR / SVLIP	APAYA / SRCTH	LGVGA / DVGCA	SMVVA / DNGEL
Langat virus	RGSRR / TTIDW	GMCLT / ATVRR	SRSRR / SVLIP	APAYA / SRCTH	LGVGA / DVGCA	SMVVA / DNGAL
Alkhurma virus	RGKRR / STTGL	TLVIS / ATIRR	GRSRR / SVSIP	APTYA / TRCTH	LGVGA / DMGCA	SMVLA / DNGAM
Kyasanur forest disease virus	RGKRR / STTGL	TLVFS / ATVRR	GRNRR / SVSIP	APTYA / TRCTH	LGVGA / DMGCA	SMVLA / DNGAM
Powassan virus	RGRRR / SGVDW	TMAMA / TTIHR	SRGKR / SVVIP	GPVYA / TRCTH	MGVGA / DYGCA	SMVVA / DNGAL
Deer tick virus	RGRRR / SGVDW	AMAMA / TSIHR	FRGRR / SVVIP	GPVYA / TRCTH	MGVGA / DYGCA	SMVMA / DNGAM
Kadam virus	NGKRK / SAGGG	SVAGA / AVVVQ	RRQTR / SVHIT	APAYA / SRCVH	LGVGA / DIGCA	SQVLA / FNDGL
Meaban virus	GRNRR / GGGSF	AIIAG / AVLME	KRHRR / ELSIS	VPAYA / SRCVH	LGVGA / DYGCA	STVLA / MTDGF
Saumarez Reef virus	GRNRR / GPGGM	GGCMG / ALVMT	SRARR / SISIT	VPAYA / SKCVH	LGVGA / DYGCA	STVLA / MTDGL
Apoi virus	KGGRR / GGKSV	PIALS / AVVMN	TRTRR / DVTIQ	APAYA / STCVS	TGVVG / EIGCM	GLVMA / FDEEP
Rio Bravo virus	KKQRR / GGTES	TGLMA / MQVSQ	HRLKR / SLSIT	APSYS / TQCVN	TGVMG / DHGCA	GLVYA / GSMTA
Zika virus	ERKRR / GADTS	TTAMA / AEITR	RRSRR / AVTLP	APAYS / IRCIG	TAVSA / DVGCS	SMVTA / GSTDH
West Nile virus	KQKKR / GGKTG	ASVGA / VTLSN	RRSRR / SLTVQ	APAYS / FNCLG	VNVHA / DTGCA	SQVNA / YNADM
Yellow fever virus	SRKRR / SHDVL	LMTGG / VTLVR	RRSRR / AIDLP	GPAYS / AHCIG	LGVGA / DQGCA	SWVTA / GEIHA
Yokose virus	KRKRR / SSVSC	VTVGA / LQIGR	RRNRR / SVALT	APAYS / THCTN	TGVGA / EQACA	SWVSA / GEGRM
Cell fusing agent virus	LESRR / TTGDP	VLCGC / VVIDM	KREKR / SREPP	TTVKG / EFVEP	YYVRA / DLGCG	GKANA / QSDFR
Kamiti River virus	LEKQR / SGPNL	GLCYG / EMLRY	VRRRR / APQPQ	NVVKA / SSIEP	RSVSA / DVGCG	GKAHA / CSDFR
Tamana bat virus	QKRQK / SSGGY	MVIFC / GYQSG	HRTRR / SVTET	ILVIA / QFYLAD	EVVAA / DKYVL	NVVKA / SKMNK

Virus	Cleavage sites (protease)					
	NS2A/NS2B (VSP)	NS2B/NS3 (VSP)	NS3/NS4A (VSP)	NS4A/2K (VSP)	2K/NS4B (Signalase)	NS4B/NS5 (VSP)
Mpulungu flavivirus	RAQRR / AALGD	PGNRR / ADLVG	QALRR / SAGDI	PGGQR / GVLDN	GLVYC / NEMGL	RDGRR / GAGHM
Ngoye virus	-	-	HALRR / SAGDI	PGGQR / GVLDN	GMVYC / NEMGL	RDGRR / GAGHM
Louping ill virus	HRGRR / SFSEP	RSSRR / SDLVY	ASGRR / SFGDV	TGKQR / SSDDN	GLVAA / NEMGF	SGGRR / GGSDG
Tick borne encephalitis virus	HRGRR / SFSEP	RSSRR / SDLVF	ASGRR / SFGDV	AGKQR / SSDDN	GLVAA / NEMGF	SGGRR / GGSEG
Omsk hemorrhagic fever virus	RRDRR / SFSEP	RSARR / SDLVF	ASGRR / SLGDM	AGKQR / SSDDN	GLVAA / NEMGF	SGTRR / GGSEG
Langat virus	SRGRR / SFNEP	GSPRR / TDLVF	ASGRR / SVGDV	TGKQR / SSDDN	GMVAA / NEMGL	TGTRR / GGSEG
Alkhurma virus	RRNRR / SFSEP	SSGRR / SELVF	ASGRR / SVGDV	PGKQR / SSDDN	GLVTA / NEMGM	TGTRR / GGADG
Kyasanur forest disease virus	RRNRR / SFSEP	GSGRR / SELVF	ASGRR / SVGDV	PGKQR / SSDDN	GLVTA / NEMGM	TGTRR / GGAEG
Powassan virus	GRGRR / SLSEP	SSTRR / TDLVF	ASGRR / SAVDI	PGKQR / SGEDN	GLVAA / NELGY	QGARR / GGAEG
Deer tick virus	GRGRR / SLSEP	SSARR / TDLVF	ASGRR / SAMDI	PGKQR / SGEDN	GLLAA / NELGY	QGARR / GGAEG
Kadam virus	FGKQR / SLEEP	SRNRR / SDLIF	AESRK / SASLV	PGTQR / SGEDT	GAVAA / NELGW	GPTRR / GLTGG
Meaban virus	HGNKR / SVSDA	GSERR / SDLVW	AEGRR / GVGRG	PGTQR / SFEDN	GLVAA / NEMGW	ARDRR / GPGST
Saumarez Reef virus	RSGRR / SVADA	NDTRR / GDLVW	AEGRR / SIGGG	PGTQR / SFEDN	GIVAA / NEMGW	QRDRR / GPGSA
Apoi virus	RSGQR / SVDPI	RSIQK / SNTSF	AKGKR / SGMTI	EGMQR / TQVDS	AAVVA / NEMGF	SENRR / GVSSS
Rio Bravo virus	HRGQR / ATDYT	DATQR / SIIVF	AQMRR / SGVLL	EGMQR / TQIDS	VTVVA / NEMRL	RSDRR / GIVTS
Zika virus	RSGKR / SWPPS	KTGKR / SGALW	AAGKR / GAALG	PEKQR / SPQDN	GLITA / NELGW	LVKRR / GGGTG
West Nile virus	PNRKR / GWPAT	QYTKR / GGVLW	ASGKR / SQIGL	PEKQR / SQTDN	SAVAA / NEMGW	PGLKR / GGAKG
Yellow fever virus	IFGRR / SIPVN	RGARR / SGDVL	AEGRR / GAAEV	PGQQR / SIQDN	SAVAA / NELGM	KTGRR / GSANG
Yokose virus	NGKVR / SIDWT	YTKQR / SNILW	ATTTR / SITAV	TGMQR / SIQDN	ALIVA / NENGY	QANRR / GGTGS
Cell fusing agent virus	NI	TANNR / SDDLL	WETRK / VSIDF	SIGNR / SYMDS	CGVLA / WEMRM	NQFRA / LEKST
Kamiti River virus	NI	SEQNR / SDDLL	WDTRK / LSIEF	AIGNR / SYMDS	CGVLA / WEMRL	NQFRA / LEKST
Tamana bat virus	NI	NLRDK / SKGLI	PLVQR / VFSGI	GITQR / EKSTG	YYILA / DGEIL	KTTQR / FRSSI

NI: Not identified, VSP: viral serine protease

Supplementary Table S4. Flavivirus reference sequences used for compositional analyses in this study

Group	Accession	Flavivirus	Seq. length	Reference
ISFV	AB488408	Aedes flavivirus	11064	[2]
ISFV	KX148546	Anopheles flavivirus variant 1	10588	[3]
ISFV	KX148547	Anopheles flavivirus variant 2	10529	[3]
ISFV	KX669689	Calbertado virus	10733	[4]
ISFV	AB377213	Culex flavivirus	10837	[2]
ISFV	HE574574	Culex theileri flavivirus RP 2011	10538	[2]
ISFV	KT599442	Culiseta flavivirus	10864	[5]
ISFV	M91671	Flavivirus sp.	10695	[2]
ISFV	JQ268258	Hanko virus	10158	[2]
ISFV	AY149905	Kamiti River virus	11375	[2]
ISFV	KY320649	La Tina virus	10050	[4]
ISFV	KX907452	Menghai flavivirus	10897	[6]
ISFV	KP688058	Mercadeo virus	10938	[2]
ISFV	KC464457	Mosquito flavivirus	10865	[2]
ISFV	AB981186	Mosquito flavivirus	10863	[2]
ISFV	JQ957875	Nienokoue virus	10878	[7]
ISFV	KY345399	Ochlerotatus caspius flavivirus	10370	[8]
ISFV	KC505248	Palm Creek virus	10095	[2]
ISFV	KT192549	Parramatta River virus	10893	[2]
ISFV	FJ644291	Quang Binh virus	10865	[2]
ISFV	MH899446	Sabethes flavivirus	10585	[9]
ISFV	KU201526	Xishuangbanna aedes flavivirus	10884	[10]
ISFV	GQ165809	Nakiwogo virus	10122	[2]
ISFV	KX245154	Cuacua virus	9747	[11]
MBFV	AY898809	Alfuy virus	10960	[2]
MBFV	KF917535	Aroa virus	10248	[2]
MBFV	HQ644143	Bagaza virus	10794	[2]
MBFV	KM225264	Bainyik virus	10648	[2]
MBFV	JF312912	Baiyangdian virus	10278	[2]
MBFV	KU308380	Bamaga virus	10203	[12]
MBFV	DQ859056	Banzi virus	10182	[2]
MBFV	DQ859057	Bouboui virus	10173	[2]
MBFV	AY632536	Bussuquara virus	10815	[2]
MBFV	KF917536	Cacipacore virus	10284	[2]
MBFV	JQ308185	Chaoyang virus	10733	[2]
MBFV	KC692512	Dengue virus 1	10735	[2]
MBFV	KC294208	Dengue virus 2	10723	[2]
MBFV	KF973479	Dengue virus 3	10569	[2]

MBFV	JN983813	Dengue virus 4	10649	[2]
MBFV	JQ086551	Donggang virus	10791	[2]
MBFV	JQ920422	Duck egg drop syndrome virus	10990	[2]
MBFV	KF573582	Duck flavivirus KPS54A61/THA	10990	[2]
MBFV	KC990542	Duck Tembusu virus	10990	[2]
MBFV	DQ859060	Edge Hill virus	10206	[2]
MBFV	AY632537	Entebbe bat virus	10236	[2]
MBFV	KM361634	Fitzroy River virus	10807	[13]
MBFV	AY632538	Iguape virus	10922	[2]
MBFV	AY632539	Ilheus virus	10755	[2]
MBFV	KC692067	Ilomantsi virus	10365	[2]
MBFV	KC734550	Israel turkey meningoencephalomyelitis virus	10792	[2]
MBFV	AF254452	Japanese encephalitis virus	10970	[2]
MBFV	DQ859066	Jugra virus	10173	[2]
MBFV	AY632540	Kedougou virus	10723	[2]
MBFV	AY632541	Kokobera virus	10874	[2]
MBFV	EU082200	Koutango virus	10302	[2]
MBFV	KT934798	Kunjin virus	10302	[2]
MBFV	FJ606789	Lammi virus	10727	[2]
MBFV	AF161266	Murray Valley encephalitis virus	11014	[2]
MBFV	KF917538	Naranjal virus	10290	[2]
MBFV	KC788512	New Mapoon virus	10864	[2]
MBFV	KJ210048	Nhumirim virus	10891	[2]
MBFV	EU159426	Nounane virus	10755	[2]
MBFV	JX236040	Ntaya virus	10943	[2]
MBFV	KJ152564	Paraiso Escondido virus	10761	[2]
MBFV	DQ859067	Potiskum virus	10173	[2]
MBFV	AY632542	Rocio virus	10794	[2]
MBFV	DQ859062	Saboya virus	10173	[2]
MBFV	DQ837642	Sepik virus	10793	[2]
MBFV	JX477686	Sitiawan virus	10278	[2]
MBFV	KF917541	Sokoluk virus	10242	[2]
MBFV	DQ859064	Spondweni virus	10290	[2]
MBFV	DQ525916	St. Louis encephalitis virus	10940	[2]
MBFV	KF917540	Stratford virus	10245	[2]
MBFV	EU879061	T'Ho virus	10937	[14]
MBFV	KM225265	Torres virus	10735	[2]
MBFV	DQ859065	Uganda S virus	10182	[2]
MBFV	KJ438721	Usutu virus	11065	[2]
MBFV	EU707555	Wesselsbron virus	10814	[2]
MBFV	DQ080055	West Nile virus	11018	[2]

MBFV	EU082199	Yaounde virus	10293	[2]
MBFV	JN811142	Yellow fever virus	10862	[2]
MBFV	AB114858	Yokose virus	10857	[2]
MBFV	KF268948	Zika virus	10788	[2]
MBFV	EU078325	Barkedji virus	10582	[2]
MBFV	KY320648	Kampung Karu virus	10311	[4]
MBFV	KY290249	Long Pine Key virus	10380	[4]
MBFV	MF139576	Marisma mosquito virus	10848	[4]
MBFV	MF139575	Nanay virus	10804	[4]
MBFV	KJ152564	Paraiso Escondido virus	10761	[2]
NKV	AF160193	Apoi virus	10116	[2]
NKV	KJ469370	Batu Cave virus	10131	[2]
NKV	KJ469371	Jutiapa virus	10125	[2]
NKV	AJ242984	Modoc virus	10600	[2]
NKV	AJ299445	Montana myotis leukoencephalitis virus	10690	[2]
NKV	KJ469372	Phnom Penh bat virus	10131	[2]
NKV	AF144692	Rio Bravo virus	10140	[2]
NKV	AF285080	Tamana bat virus	10053	[15]
TBFV	JF416955	Alkhumra hemorrhagic fever virus	10749	[2]
TBFV	AF311056	Deer tick virus	10800	[2]
TBFV	DQ235145	Gadgets Gully virus	10251	[2]
TBFV	DQ235153	Greek goat encephalitis virus	10245	[2]
TBFV	DQ235146	Kadam virus	10215	[2]
TBFV	KF815940	Kama virus	10688	[2]
TBFV	AY863002	Karshi virus	10653	[2]
TBFV	JF416958	Kyasanur forest disease virus	10774	[2]
TBFV	EU790644	Langat virus	10943	[2]
TBFV	KP144331	Louping ill virus	10880	[2]
TBFV	DQ235144	Meaban virus	10266	[2]
TBFV	KT224355	Negishi virus	10605	[2]
TBFV	AY193805	Omsk hemorrhagic fever virus	10787	[2]
TBFV	KT224351	Powassan virus	10742	[2]
TBFV	DQ235149	Royal Farm virus	10254	[2]
TBFV	DQ235150	Saumarez Reef virus	10269	[2]
TBFV	KP144332	Spanish goat encephalitis virus	10870	[2]
TBFV	DQ235152	Spanish sheep encephalitis virus	10245	[2]
TBFV	U27491	Tick borne encephalitis virus	11141	[2]
TBFV	DQ235151	Turkish sheep encephalitis virus	10245	[2]
TBFV	KF815939	Tyuleniy virus	10673	[2]
Marine	MK473878	Crangon crangon flavivirus	11434	[16]
Marine	MF776369	Cyclopterus lumpus virus	11454	[17]

Marine	MK473877	Gammarus chevreuxi flavivirus	11231	[16]
Marine	MK473881	Gammarus pulex flavivirus	11883	[16]
Marine	MK473875	Southern pygmy squid flavivirus	12567	[16]
Marine	MG599986	Wenzhou shark flavivirus	10670	[18]

Abbreviations: TBFV, tick-borne flavivirus; MBFV, mosquito-borne flavivirus; ISFV, insect-specific flavivirus; NKV, non-known vector flavivirus.

Supplementary Table S5. Synthesized RNA used in this study

RNA	Sequence (5'-3')	Length (mer)
(+31)-xrRNA1	uauagauauuaaauaaugauaccacaacaaaCCUCGUGGGGUGAGCC CAAAAGCAGGAAGAGCUGACCGUCUUCCACCCCAACG CUCUAGGGAAGGUCAACAACGGCUGAGGCCGUU	117
(+31)-xrRNA2	cuaacgcgcagaacaaaacagaaacauaaaaAGGGUGAGCCUGUAUA GCAGAAAGAACUAACCGUCUUCCACCCUAAAGUACAG GAAGGUUAAUCAAAGGGUGAGCCUGAGUAC	114
5′-PO_4 31-mer control RNA	UAUAGAUAUUAAAUAAUGAUACCACAACAAA	31

Small letters indicate the 31-mer additional leader sequences for Xrn1 resistance assay

Reference

1 Patel, P. *et al.* Development of one-step quantitative reverse transcription PCR for the rapid detection of flaviviruses. *Virol J* **10**, 58, doi:10.1186/1743-422X-10-58 (2013).

2 Simón, D., Fajardo, A., Sóñora, M., Delfraro, A. & Musto, H. Host influence in the genomic composition of flaviviruses: A multivariate approach. *Biochem Biophys Res Commun* **492**, 572-578, doi:10.1016/j.bbrc.2017.06.088 (2017).

3 Fauver, J. R. *et al.* West African Anopheles gambiae mosquitoes harbor a taxonomically diverse virome including new insect-specific flaviviruses, mononegaviruses, and totiviruses. *Virology* **498**, 288-299, doi:10.1016/j.virol.2016.07.031 (2016).

4 Ochsenreiter, R., Hofacker, I. L. & Wolfinger, M. T. Functional RNA Structures in the 3'UTR of Tick-Borne, Insect-Specific and No-Known-Vector Flaviviruses. *Viruses* **11**, doi:10.3390/v11030298 (2019).

5 Misencik, M. J., Grubaugh, N. D., Andreadis, T. G., Ebel, G. D. & Armstrong, P. M. Isolation of a Novel Insect-Specific Flavivirus from Culiseta melanura in the Northeastern United States. *Vector Borne Zoonotic Dis* **16**, 181-190, doi:10.1089/vbz.2015.1889 (2016).

6 Zhang, X. *et al.* Complete genome sequence of Menghai flavivirus, a novel insect-specific flavivirus from China. *Arch Virol* **162**, 1435-1439, doi:10.1007/s00705-017-3232-5 (2017).

7 Junglen, S. *et al.* Host Range Restriction of Insect-Specific Flaviviruses Occurs at Several Levels of the Viral Life Cycle. *mSphere* **2**, doi:10.1128/mSphere.00375-16 (2017).

8 Ergünay, K. *et al.* Co-circulation of West Nile virus and distinct insect-specific flaviviruses in Turkey. *Parasit Vectors* **10**, 149, doi:10.1186/s13071-017-2087-7 (2017).

9 Gravina, H. D. *et al.* Identification of insect-specific flaviviruses in areas of Brazil and Paraguay experiencing

endemic arbovirus transmission and the description of a novel flavivirus infecting Sabethes belisarioi. *Virology* **527**, 98-106, doi:10.1016/j.virol.2018.11.008 (2019).

10 Fan, H. *et al.* Complete genome sequence of Xishuangbanna flavivirus, a novel mosquito-specific flavivirus from China. *Arch Virol* **161**, 1723-1727, doi:10.1007/s00705-016-2827-6 (2016).

11 Cholleti, H. *et al.* Discovery of Novel Viruses in Mosquitoes from the Zambezi Valley of Mozambique. *PLoS One* **11**, e0162751, doi:10.1371/journal.pone.0162751 (2016).

12 Colmant, A. M. G. *et al.* A newly discovered flavivirus in the yellow fever virus group displays restricted replication in vertebrates. *J Gen Virol* **97**, 1087-1093, doi:10.1099/jgv.0.000430 (2016).

13 Johansen, C. A. *et al.* Characterization of Fitzroy River Virus and Serologic Evidence of Human and Animal Infection. *Emerg Infect Dis* **23**, 1289-1299, doi:10.3201/eid2308.161440 (2017).

14 Briese, T. *et al.* Complete genome sequence of T'Ho virus, a novel putative flavivirus from the Yucatan Peninsula of Mexico. *Virol J* **14**, 110, doi:10.1186/s12985-017-0777-6 (2017).

15 de Lamballerie, X. *et al.* Genome sequence analysis of Tamana bat virus and its relationship with the genus Flavivirus. *J Gen Virol* **83**, 2443-2454, doi:10.1099/0022-1317-83-10-2443 (2002).

16 Parry, R. & Asgari, S. Discovery of Novel Crustacean and Cephalopod Flaviviruses: Insights into the Evolution and Circulation of Flaviviruses between Marine Invertebrate and Vertebrate Hosts. *J Virol* **93**, doi:10.1128/JVI.00432-19 (2019).

17 Skoge, R. H., Brattespe, J., Økland, A. L., Plarre, H. & Nylund, A. New virus of the family Flaviviridae detected in lumpfish (Cyclopterus lumpus). *Arch Virol* **163**, 679-685, doi:10.1007/s00705-017-3643-3 (2018).

18 Shi, M. *et al.* The evolutionary history of vertebrate RNA viruses. *Nature* **556**, 197-202, doi:10.1038/s41586-018-0012-7 (2018).

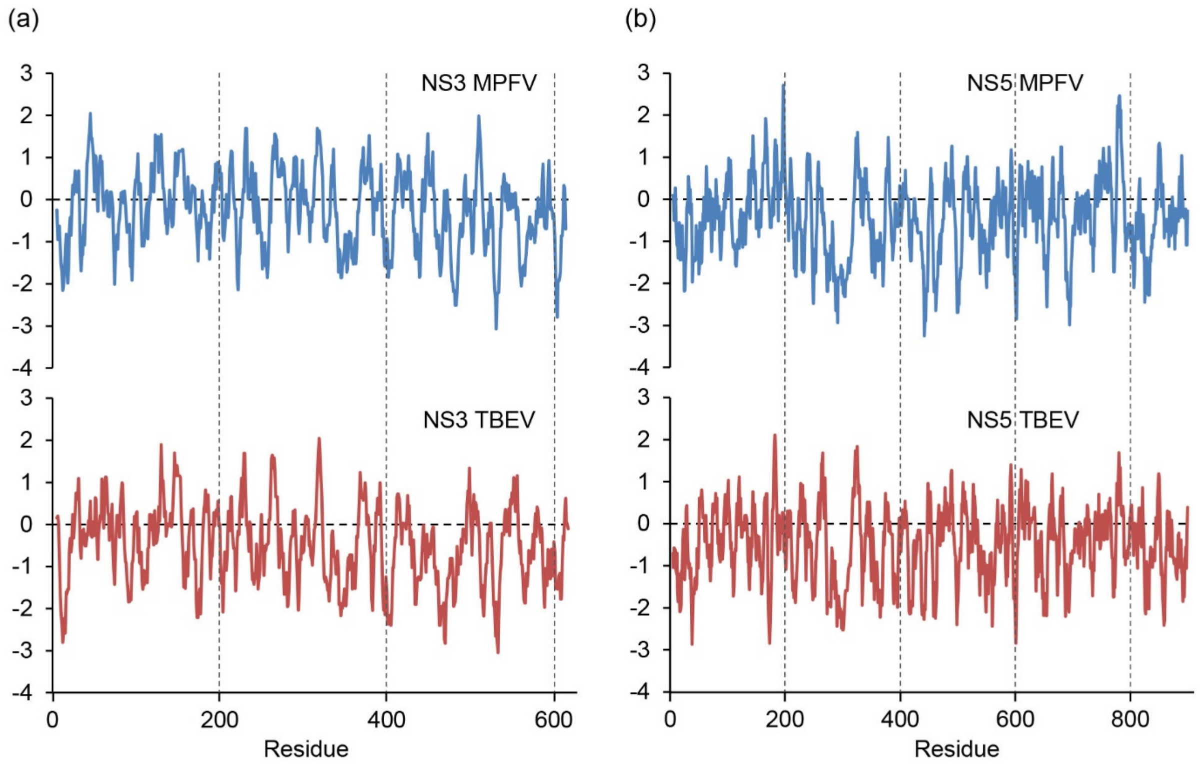

Fig. S1

Comparison analyses of hydropathy profile. Amino acid hydropathy values of NS3 (a) and NS5 (b) were determined by Kyte and Doolittle scale. Those of Mpulungu flavivirus (MPFV) were compared with tick-borne encephalitis virus (TBEV). Negative scores mean a hydrophilic domain. A sliding window size of nine amino acids.

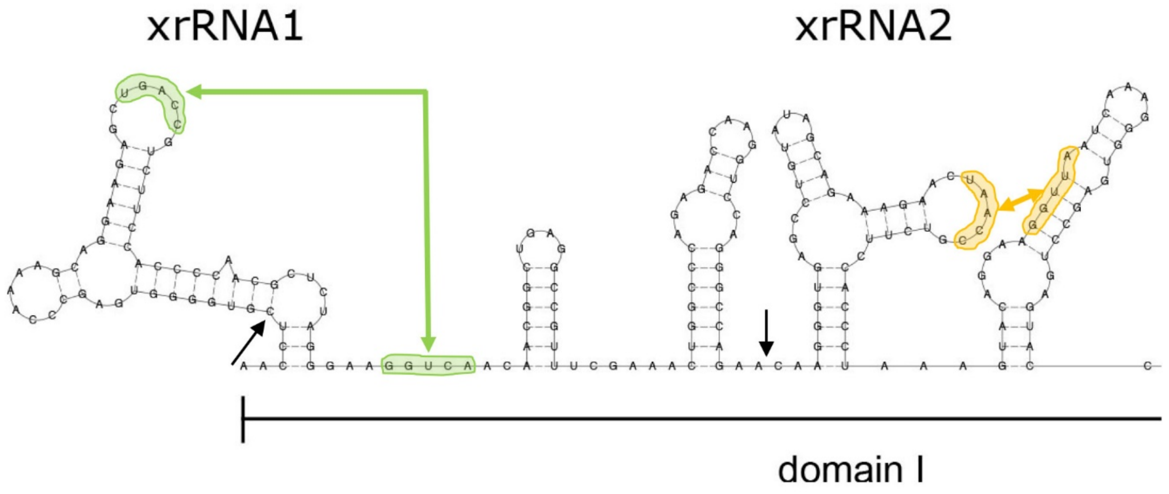

Fig. S2

Characterization of xrRNA1 and xrRNA2 derived from Mpulungu flavivirus (MPFV). Secondary structures of relevant parts of the 3′-UTR of MPFV. The mapped Xrn1 halt sites is indicated with black arrows. Green and orange arrows indicate potential pseudoknot interactions between complementary regions in xrRNA1 and xrRNA2, respectively.

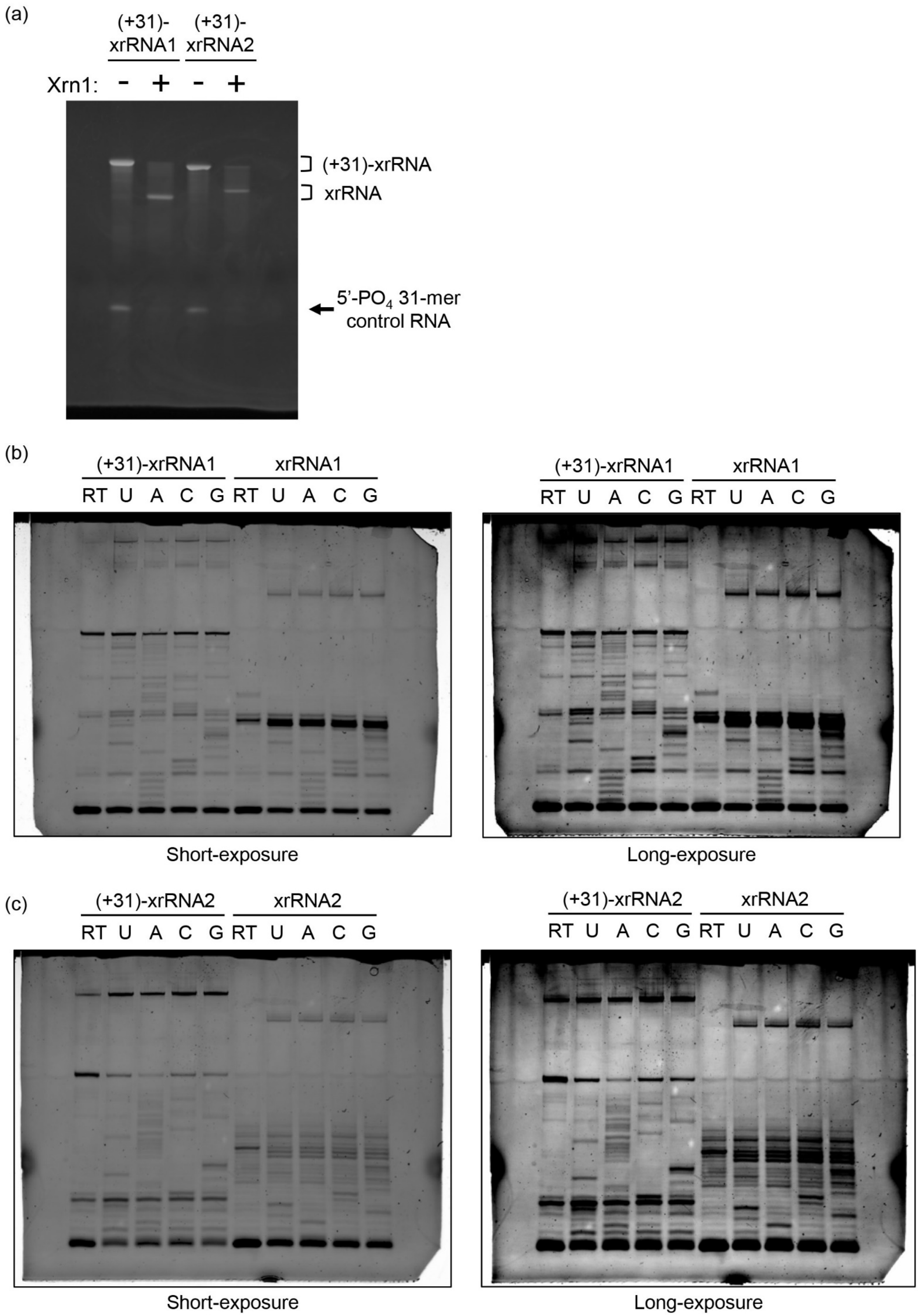

(a)
(+31)-xrRNA1
(+31)-xrRNA2
Xrn1: - + - +
(+31)-xrRNA
xrRNA
5'-PO4 31-mer control RNA
(b)
(+31)-xrRNA1
xrRNA1
RT U A C G RT U A C G
Short-exposure
(+31)-xrRNA1
xrRNA1
RT U A C G RT U A C G
Long-exposure
(c)
(+31)-xrRNA2
xrRNA2
RT U A C G RT U A C G
Short-exposure
(+31)-xrRNA2
xrRNA2
RT U A C G RT U A C G
Long-exposure

Fig. S3

Images of original full-length gels partly represented in Fig. 6c, 6d, and 6e. Images of full-length gels are represented in both short and long exposure conditions (b, c).
